# Black Poverty Leads White Americans to Blame Racial Inequality on Black Americans Themselves

**DOI:** 10.1177/19485506251329122

**Published:** 2025-04-03

**Authors:** Nicolas Sommet, B. Keith Payne

**Affiliations:** 1 University of Lausanne, Switzerland; 2 The University of North Carolina at Chapel Hill, USA

**Keywords:** race, racial inequality, Black poverty, attributions of racial inequality, racial equity policies, interracial anxiety

## Abstract

In this article, we argue that White Americans exposed to Black poverty may rationalize racial inequality in ways that deflect blame from their own racial group. In Study 1 (11,855 participants; 537 counties), White Americans in counties with higher Black poverty rates were ironically more likely to believe in racial equality of opportunity, while Black residents were less likely. In Study 2 (4,297 participants; 227 counties), White Americans living in counties with higher Black poverty attributed racial inequality to more internal causes (lack of effort), which predicted reduced support for racial equity policies. In Study 3 (1,036 participants), White Americans experimentally exposed to Black poverty made more internal attributions, an effect mediated by increased interracial anxiety and identity threat. Effects on external attributions were inconsistent across studies. We discuss how internal attributions function as a psychological tool for White Americans to deflect negative emotions and maintain a positive group identity.

More than one-fifth of Black Americans live below the poverty line, twice as many as White Americans (U.S. Census Bureau, 2022). Although this national statistic is noteworthy on its own, it obscures important regional variations: In the quintile of counties with the lowest rates of Black poverty, fewer than 10% of Black Americans are poor, whereas in the quintile with the highest rates, more than 40% of Black Americans are poor (Figure 1).

**Figure 1:**
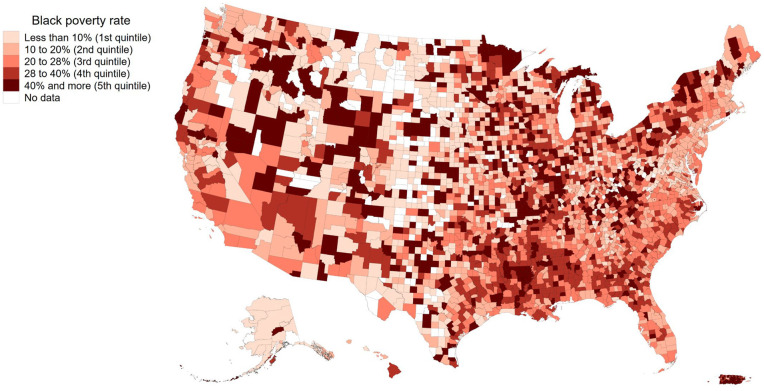
Spatial Distribution of Black Poverty Across U.S. Counties *Note.* Quintile classification was used for the data representation. The map was constructed using the most recent available data from the U.S. Census Bureau (i.e., 2021) and the Stata user-written command *spmap* (Pisati, 2018).

Sociologists studying the spatial distribution of opportunities have shown that these variations are not random but reflect systemic inequalities rooted in history (e.g., Baker, 2022). For example, counties with larger concentrations of enslaved individuals prior to the Civil War have higher Black poverty rates today (O’Connell, 2020). Similarly, cities once considered “sundown towns” (O’Connell, 2019), and places where redlining and related practices created segregated schools and neighborhoods (Rothstein, 2017) exhibit higher Black poverty rates today. The structural barriers established during these historical periods, such as unequal access to resources and education, persist in contemporary society.

But how do White Americans in areas with high Black poverty interpret racial inequality? A naïve prediction suggests that they would attribute racial inequality to external forces, given their chronic exposure to the enduring legacy of systemic racial inequalities. However, in this research, we propose a hypothesis informed by psychological theory that predicts the opposite: Contexts with greater Black poverty ironically lead White Americans to place more blame for racial inequality on Black Americans themselves.

## Attributions of Racial Inequality Among White Americans

Research suggests that racial inequality can be attributed to either internal or external factors (for pioneering work, see Hewstone, 1990; Kluegel & Smith, 1986/2017; Sniderman et al., 1986). *Internal attributions* explain racial disparities by factors such as a lack of will and effort by Black Americans (Knight, 1998) or substandard skills and limited innate ability (Byrd & Ray, 2015). *External attributions* point to factors beyond the control of Black Americans, such as biased opportunity and reward structures (Sperling & Vaughan, 2009) and discrimination (Tuch & Hughes, 1996).

White Americans tend to emphasize internal causes for racial inequality. Most White Americans believe in racial equality of opportunity (e.g., in loan applications, employment) and reject the idea that discrimination or institutional hurdles explain why many Black Americans struggle to get ahead (Pew Research Center, 2016). The most common explanation for racial inequality offered by White Americans is the lack of motivation of Black Americans, narrowly surpassing the lack of educational opportunities available to Black Americans (Shelton, 2017). Such attributional patterns carry fundamental implications for racial policy attitudes (Sniderman et al., 1986). Internal attributions of racial inequality are closely associated with the beliefs that Black Americans are responsible for their own plight and do not deserve help (for a review, see Sahar, 2014). White Americans who make such attributions are less supportive of racial equity policies, including government-sponsored assistance for Black Americans or affirmative action in hiring (Bobo & Kluegel, 1993; Nelson & Joselus, 2023; Tuch & Hughes, 2011).

## Attributions of Racial Inequality in Places With High Black Poverty

Why do White Americans favor internal over external attributions of racial inequality? Research and theory suggest that internal attributions may serve a self-protective function for members of high-status groups. Social Identity Theory posits that social groups are an important source of self-esteem, which people may defend by derogating members of outgroups (Tajfel & Turner, 1979). Supporting this claim, research shows that identity-related threats lead people to stereotype outgroup members (Fein & Spencer, 1997), while self-affirmation reduces this tendency (Sherman et al., 2017). Likewise, the functionalist analysis of stereotypes suggests that stereotyping can not only help to protect one’s personal status but also to uphold the status of one’s group and legitimize the social order (Jost & Banaji, 1994). Stereotyping may function as an ideological tool to reduce dissonance for both the advantaged and disadvantaged (Jost & Hunyady, 2003). Specifically, ascribing traits such as laziness to Black individuals serves as *a function of detachment*, enabling White Americans to downplay, dismiss, or distance themselves from racial inequality (Snyder & Miene, 1994). Such internal attributions can be a way to deflect feelings of undeserved privilege (Knowles et al., 2014) and mitigate negative self-conscious emotions associated with benefiting from racial inequality (Ford et al., 2022).

Such theoretical reasoning can guide predictions about how White Americans in places with high Black poverty interpret racial inequality. In these places, mental associations between race and wealth are likely reinforced, leading White Americans to feel uneasy about being the economic beneficiaries of inequality (Brown-Iannuzzi et al., 2019). Accordingly, research indicates that White Americans living in places where Black Americans are relatively poorer perceive them as sources of threat and report heightened interracial anxiety—a sense of discomfort and worry during interracial encounters (Gordils et al., 2021, 2023). Consequently, they may be particularly motivated to dismiss the concept of racial privilege, overestimate the equality of opportunity between Black and White Americans, and reject structural explanations of racial inequality (for relevant research, see Davidai & Walker, 2022; Kraus et al., 2019; Phillips & Lowery, 2018). In other words, White Americans from locales bearing the most visible scars of the country’s historical legacy might be the most inclined to endorse the belief that racial inequality is due to individual rather than structural deficits. Furthermore, since there is no reason for Black Americans in locales with higher Black poverty to experience any anxiety or identity-related threat, they should not make more internal attributions of racial inequality.

## Overview of the Studies and Hypotheses

Three studies examined how White Americans in places with high Black poverty make sense of racial inequality. In Study 1, we analyzed Gallup’s Minority Rights and Relations Series (MRRS), a nationally representative U.S. survey with oversamples of Black and Hispanic Americans. We used beliefs in racial equality of opportunity as a proxy for attributions. We hypothesized that White Americans residing in counties with greater Black poverty hold stronger beliefs in racial equality of opportunity, whereas Black Americans do not (H_1_). We did not formulate hypotheses regarding Hispanic Americans, but we report results for this group for the sake of completeness. Two follow-up preregistered studies examined this phenomenon in greater depth while focusing on White Americans. In Study 2, we analyzed the General Social Survey (GSS), a nationally representative U.S. survey. We hypothesized that White Americans residing in counties with greater Black poverty make more internal (H_2a_) and fewer external (H_2b_) attributions of racial inequality. We additionally tested whether such an attributional pattern predicted lower support for racial equity policies (H_3_). In Study 3, we recruited a sample of 1,036 White Americans representative of the national population in terms of gender and education. We used an experimental design to test the causal effects of exposure to Black poverty on internal (H_2a_) and external (H_2b_) attributions. As we posited that internal attributions arise as a response to the interracial anxiety and identity threat elicited by Black poverty (Abadie et al., 2023; Dovidio et al., 2017; Nelson, 1999), we also tested whether these variables mediated the effects (H_4_). Table 1 summarizes the hypotheses and the main conclusions drawn from the empirical findings.

**Table 1:** Summary of the Hypotheses and the Main Conclusions Drawn From the Empirical Findings

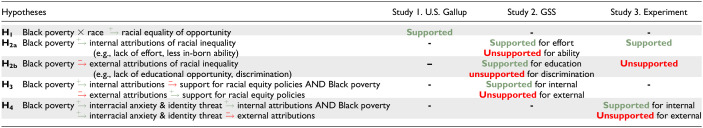

*Note.*^**+**^**→** means “positive effect” and ^–^**→** means “negative effect.” A dash (“–”) indicates that the hypothesis was not tested in the study.

## Study 1. U.S. Gallup. Black Poverty and Beliefs in Racial Equality of Opportunity

Study 1 used Gallup’s MRRS to test whether White Americans (vs. Black Americans) residing in counties with greater Black poverty report stronger beliefs in racial equality of opportunity. The study was not preregistered.

### Method

#### Transparency and Openness

For all studies, we report all data exclusions, manipulations, and measures. Preregistrations, materials, data (or instructions to access survey data), scripts, and log files are available at: https://osf.io/n5w2t.

#### Sample

Gallup’s MRRS used probability sampling and oversampled Black and Hispanic Americans. We used the five waves that included county identifiers and measures of beliefs in racial equality of opportunity. Counties served as the higher-level units of analysis because they are the smallest cluster for which the U.S. Census Bureau provides annual poverty estimates. We included participants with nonmissing responses on the outcome (98.7%). The sample comprised 11,855 participants from 537 counties: 50% non-Hispanic White, 28% non-Hispanic Black, and 22% Hispanic Americans (see Supplementary Table S1 for sample characteristics). Asian Americans (*n* = 372) and participants from nonspecified groups (*n* = 358) were excluded due to their limited number. This sample size was sufficient to detect a fully attenuated interaction involving a small-sized simple effect of Black poverty among White participants (*r* = .10) and a null effect among Black participants (*r* = .00) with *power* = .95 (for the simulation-based sensitivity analysis, see Supplementary Materials, p. 3).

#### Variables

##### Black Poverty

We used the 1-year annual estimates of county-level poverty rates among Black residents from the U.S. Census Bureau (2022). The measure ranged from .01 (1% of Black residents below the poverty line) to .65 (65% below the poverty line; *M* = .25, *SD* = .10).^1^

##### Beliefs in Racial Equality of Opportunity

We a priori selected the only items relevant to racial equality of opportunity. Participants indicated whether Black Americans in their community had as good a chance as White Americans to get “good education” (62.6% yes [not available in 2018]), “any housing they can afford,” (66.5% yes) and “any kind of job for which they are qualified” (56.4% yes). The intraclass correlation coefficient (ICC) was .04 (4% of the variation was attributable to between-county differences).

### Results

#### Overview of the Multilevel Logistic Analysis

We treated within-participant responses (*N* = 35,565) as nested in participants (*K* = 11,855) and counties (*L* = 537). To maximize power and account for one item missing in the last wave, we treated within-participant responses as lower-level observations rather than aggregating them (Quené & Van den Bergh, 2004). Specifically, we used multilevel logistic regression modeling (Sommet & Morselli, 2017), regressing beliefs in racial equality of opportunity on county-level Black poverty, participants’ race/ethnicity, and their interaction. We standardized Black poverty (subtracting the grand mean and dividing by the grand standard deviation) and broke race/ethnicity down into two dummy-coded variables (comparing White to Black participants and White to Hispanic participants). We included year-fixed effects (Allison, 2009) and county-level random slopes for race/ethnicity dummies (Heisig & Schaeffer, 2019). Given the complexity of the analysis, we used restrictive iterative generalized least squares (RIGLS) rather than maximum likelihood (ML) estimation (Rasbash et al., 2004) (for the multilevel regression equation, see Supplementary Materials, p. 4).

#### Findings: H_1_. Black Poverty × Race ^**+**^**→** Racial Equality of Opportunity

**Figure 2:**
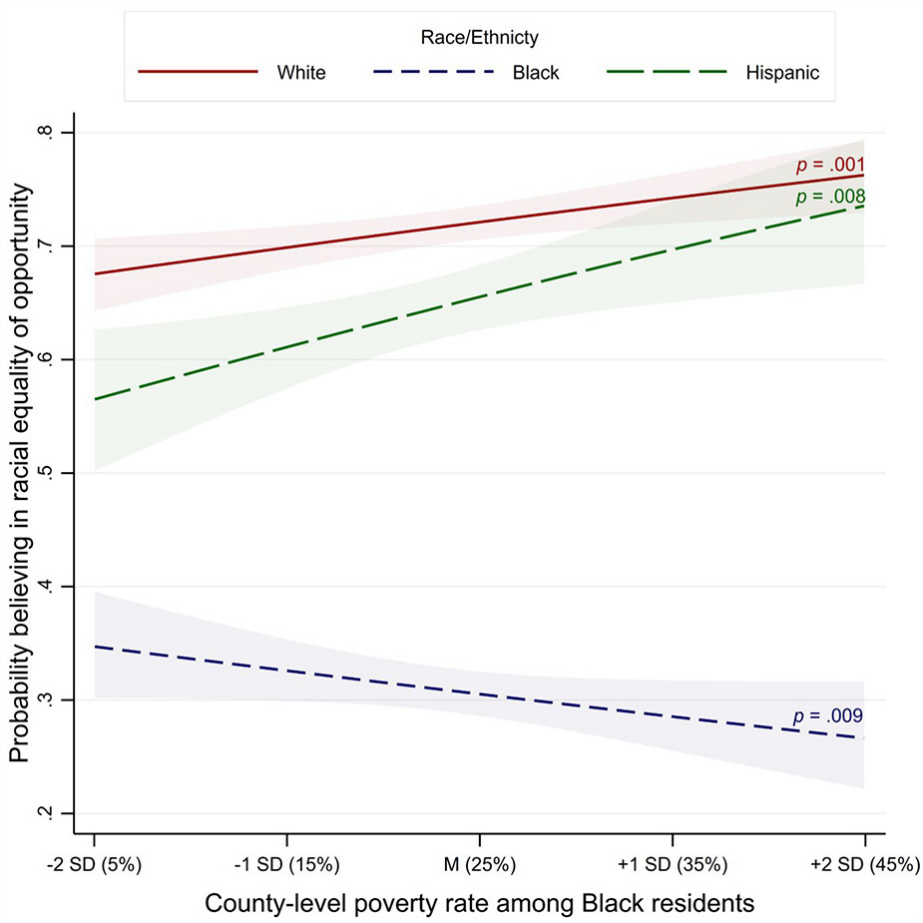
Study 1, H_1_–Focal Interaction: Probability of Believing in Racial Equality of Opportunity as a Function of Race/Ethnicity (White, Black, and Hispanic Residents) and Black Poverty (County-Level Poverty Rate Among Black Residents) *Note*. The point estimates used to build this figure were derived from a model using ML (the coefficient estimates were very similar to the main model using RIGLS); shaded areas represent 95% CIs.

Table 2 presents the full results. As shown in Figure 2, there was an interaction between Black poverty and race/ethnicity, χ^2^ (2, *K* = 11,855) = 23.38, *p* < .001. Consistent with H_1_, the effect of Black poverty differed between White and Black participants, odds ratio (*OR*) = 0.79 [0.71, 0.87], *p* < .001: When county-level Black poverty was higher by +1 *SD* (≈+10%), White residents were 13% *more* likely to hold beliefs in racial equality of opportunity, *OR* = 1.13 [1.05, 1.21], *p* = .001, whereas Black residents were 11% *less* likely to hold such beliefs, *OR* = 0.89 [0.82, 0.97], *p* = .008. The effect of Black poverty did not differ between White and Hispanic participants, *OR* = 1.04 [0.91, 1.19], *p* = .537: When county-level Black poverty was higher by +1 *SD*, Hispanic residents were 17% more likely to hold beliefs in racial equality of opportunity, *OR* = 1.17 [1.04, 1.33], *p* = .009. Controlling for an a priori-defined set of participant- and county-level sociodemographic variables, the hypothesized interaction remained significant, although the simple effect of Black poverty for White residents became nonsignificant (see Supplementary Materials, pp. 7–8).

**Table 2:** Study 1, H_1_—Full Results: ORs/95% CIs From the Model Testing the Association Between Black Poverty and Beliefs in Racial Equality of Opportunity as a Function of Race/Ethnicity

	Model terms	*OR*	95% CI
Focal variables	Intercept, *B*_000_	3.90***	[3.47, 4.39]
	County-level Black poverty (standardized), *B*_001_	1.13**	[1.05, 1.21]
	Race/Ethnicity: Dummy #1 (White [baseline] vs. Black), *B*_010_	0.18***	[0.16, 0.19]
	Race/Ethnicity: Dummy #2 (White [baseline] vs. Hispanic), *B*_020_	0.69***	[0.61, 0.77]
	Interaction: Black poverty × Dummy #1, *B*_011_	0.79***	[0.71, 0.87]
	Interaction: Black poverty × Dummy #2, *B*_021_	1.04	[0.91, 1.19]
Year fixed effects	Year 2007 (baseline) vs. 2008, δ_1_	1.14	[0.99, 1.31]
	2013, δ_2_	1.17**	[1.04, 1.32]
	2015, δ_3_	1.03	[0.90, 1.17]
	2018, δ_4_	0.23***	[0.21, 0.26]
Variance parameters	Level-2 random intercept variance, var(*u*_0jk_)	1.68	
	Level-3 random intercept variance, var(*v*_00k_)	0.22	
	Level-3 random slope variance for Dummy #1, var(*v*_01k_)	0.05	
	Level-3 random slope variance for Dummy #2, var(*v*_02k_)	0.16	
	Covariance parameter #1, cov(*u*_0jk_, *v*_01k_)	−0.12	
	Covariance parameter #2, cov(*u*_0jk_, *v*_02k_)	−0.10	

** *p* < .01. ***p < .001

## Study 2. GSS. Black Poverty, Attributions of Racial Inequality, and Support for Racial Equity Policies

Study 1 revealed that county-level Black poverty predicted stronger beliefs in racial equality of opportunity among White and Hispanic Americans, but not Black Americans. Study 2 used the GSS to further examine the ironic effects of Black poverty among White Americans by (1) using more fine-grained attribution measures and (2) investigating downstream effects on political attitudes. The study was preregistered (https://osf.io/vne8y).

### Method

#### Sample

The GSS used probability sampling to achieve representativeness. We used the six waves that included county identifiers and a module on attributions of racial inequality. As preregistered, we included participants with nonmissing responses on the outcomes (91.4%) and who identified as White (73.8%). We focused on White respondents because the number of Black and Hispanic respondents was insufficient to test race/ethnicity as moderators. The sample comprised 4,297 participants from 227 counties (see Supplementary Table S1 for sample characteristics). This sample size was sufficient to detect four small-sized main effects of Black poverty (*r*s = .10) with *power* = .95 (for the sensitivity analysis, see Supplementary Materials, p. 9).

#### Variables

##### Black Poverty

We used the same annual estimates of county-level poverty rates among Black residents as in Study 1 (*range*: .03, .57; *M* = .24, *SD* = .09).

##### Attributions of Racial Inequality

We used the four items from the module asking participants why “Black Americans have [on average] worse jobs, income, and housing than White people.” Two items measured two forms of *internal* attributions: (a) “Black Americans just don’t have the motivation or will power” (46.7% yes) and (b) “Black Americans have less in-born ability to learn” (8.0% yes). Two other items measured two forms of *external* attributions: (a) “Black Americans don’t have the chance for education” (46.9% yes) and (b) “discrimination” (32.9% yes). The ICCs ranged from .02 to .06. As preregistered, the items were treated as separate variables.

##### Support for Racial Equity Policies

We used the four GSS items relevant to policies aiming to reduce racial inequality: (a) “Should government aid blacks?” (1 = *Government should help*; 5 = *No special treatment*), (b) “Blacks should overcome prejudice without special favors” (1 = *Agree strongly*; 5 = *Disagree strongly*), (c) “Are you for or against preferential hiring and promotion of blacks?” (1 = *Strongly favors*; 4 = *Strongly opposes*), (d) “Are we spending too much [. . .] on assistance to black” (1 = *too little*; 3 = *too much*). As preregistered, scales were converted to a five-point scale, with higher scores reflecting greater support. The items were averaged (α = .74, *M* = 2.17, *SD* = 0.98, ICC = .06).

### Results

#### Overview of the Preregistered Multilevel Analysis

We first examined the associations between Black poverty and attributions (H_2a-b_). As preregistered, we treated participants (*N* = 4,297) as nested in counties (*K* = 227). Unlike Study 1, we used two-level rather than three-level modeling because each focal outcome was a single-item measure. We again used multilevel logistic regression modeling, regressing each attribution outcome on county-level Black poverty across four models. We again standardized Black poverty and included year fixed effects.^2^ This time, we used standard ML estimation (for the multilevel regression equation, see Supplementary Materials, p. 10).

#### Findings: H_2a-b_. Black Poverty ^**+**^**→** Internal/External Attributions of Racial Inequality

**Figure 3:**
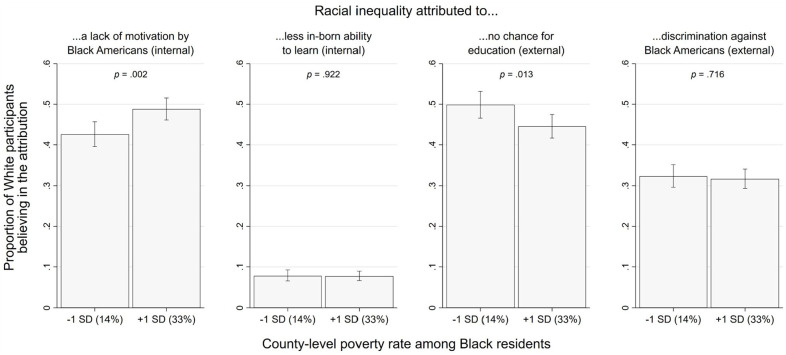
Study 1, H_2a-b_—Focal Effects: Probability of Making Internal Attributions (First and Second Panels From the Left) and External Attributions (Third and Fourth Panels From the Left) Among White Residents, as a Function of Black Poverty (County-Level Poverty Rate Among Black Residents) *Note.* Error bars represent 95% CIs.

Table 3 presents the full results, and Figure 3 shows the focal effects.

**Table 3:** Study 2, H_2-3_—Full Results: ORs/Bs and 95% CIs From the Models Testing the Association Between Black Poverty and Attributions (H_2a-b_) and the Downstream Statistical Effects on Support for Racial Equity Policies (H_3_)

		Black poverty to attributions of racial inequality (H2a-b)								Downstream effects (H3)			
		Internal attributions				External attributions				Support for racial equity policies			
		Lack of motivation (*att*_1_)		Less in-born ability (*att*_2_)		No chance for education (*att*_3_)		Discrimination (*att*_4_)		Total effect		Mediating and direct effects	
	Model terms	*OR*	95% CI	*OR*	95% CI	*OR*	95% CI	*OR*	95% CI	*B*	95% CI	*B*	95% CI
Focal variables	Black poverty (std), *B*_01_	1.13**	[1.05, 1.23]	0.99	[0.88, 1.12]	0.90*	[0.83, 0.98]	0.98	[0.91, 1.07]	−0.02	[–0.05, 0.01]	0.01	[–0.02, 0.03]
	Lack of motivation, *B*_10_											−0.42***	[–0.48, –0.37]
	Less in-born ability, *B*_20_											−0.07	[–0.16, 0.03]
	No chance for education, *B*_30_											0.45***	[0.40, 0.50]
	Discrimination, *B*_40_											0.62***	[0.56, 0.67]
Year fixed effects	2006 (baseline) vs. 2008, δ_1_	1.17	[0.92, 1.48]	1.29	[0.88, 1.88]	1.24	[0.98, 1.58]	1.15	[0.90, 1.48]	0.07	[–0.04, 0.18]	0.05	[–0.05, 0.14]
	2010, δ_2_	0.82	[0.67, 1.02]	0.99	[0.69, 1.42]	1.21	[0.98, 1.49]	1.09	[0.87, 1.36]	0.05	[–0.05, 0.15]	0.00	[–0.09, 0.08]
	2012, δ_3_	0.84	[0.67, 1.05]	0.76	[0.51, 1.13]	0.98	[0.78, 1.23]	1.10	[0.87, 1.40]	0.04	[–0.06, 0.14]	0.01	[–0.08, 0.09]
	2014, δ_3_	0.72**	[0.59, 0.89]	0.77	[0.54, 1.10]	1.00	[0.81, 1.23]	0.96	[0.77, 1.20]	0.09	[–0.00, 0.18]	0.07	[–0.01, 0.15]
	2016, δ_4_	0.69***	[0.56, 0.84]	0.75	[0.53, 1.07]	1.45***	[1.18, 1.78]	1.60***	[1.30, 1.98]	0.34***	[0.25, 0.43]	0.20***	[0.12, 0.27]
VP	Random intercept variance, var(*u*_0j_)	0.14		0.04		0.20		0.12		0.95		0.69	

*Note.* “VP” = Variance Parameters, “std” = Standardized.

* *p* < .05. **p < .01. ***p < .001.

#### Internal Attributions

Consistent with H_2a_, our analysis revealed that when county-level Black poverty was higher by +1 *SD* (≈+10%), White residents were 13% *more* likely to attribute racial inequality to a lack of motivation by Black Americans, *OR* = 1.13 [1.05, 1.23], *p* = .002. No effect was observed for attribution to less in-born ability, *OR* = 0.99 [0.88, 1.12], *p* = .929.

#### External Attributions

Consistent with H_2b_, our analysis revealed that when county-level Black poverty was higher by +1 *SD* (≈+10%), White residents were 10% *less* likely to attribute racial inequality to a lack of educational opportunity for Black Americans, *OR* = 0.90 [0.83, 0.98], *p* = .013. No effect was observed for attribution to discrimination, *OR* = 0.98 [0.91, 1.07], *p* = .716.

#### Overview of the Preregistered Multilevel Analysis

We investigated the indirect effects of Black poverty (via attributions) on support for racial equity policies (H_3_). We used multilevel *linear* regression, first regressing support for racial equity policies on county-level Black poverty, and then entering all four attribution scores as additional regressors. We again standardized Black poverty and included year fixed effects. As preregistered, we calculated indirect effects using a Monte Carlo approach with 10^7^ repetitions (Tofighi & MacKinnon, 2011) (for the multilevel regression equation, see Supplementary Materials, p. 10).

#### Findings: H_3_. Black Poverty ^**+**^**→** Attributions ^**+**^**→** Support for Racial Equity Policies

**Figure 4:**
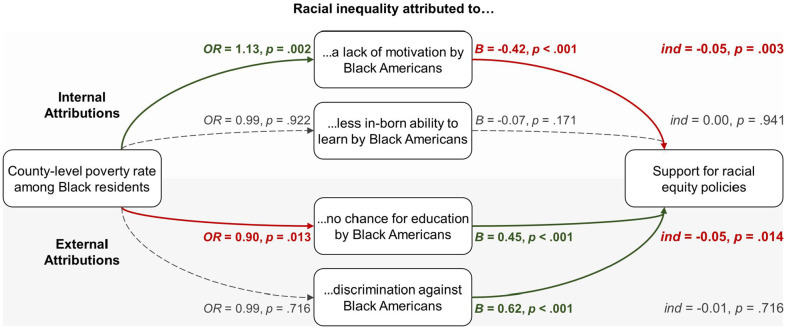
Study 1, H_3_—Paths of Interest: Indirect Effects of Black Poverty (County-Level Poverty Rate Among Black Residents) on Support for Racial Equity Policies via Internal Attributions (Upper Part) and External Attributions (Lower Part) of Racial Inequality Among White Residents *Note*. Thick green lines represent the positive effects; thick red lines represent the negative effects; dashed gray lines represent the null effects; total and direct effects are part of the model but not shown.

Table 3 presents the full results, and Figure 4 shows the paths of interest.

#### Internal Attributions

Consistent with H_3_, our analysis revealed an indirect effect of Black poverty via our first internal attribution measure, *ind* = −0.05 [−0.09, −0.02], *p* = .003. Black poverty positively predicted attribution of racial inequality to a lack of motivation, which negatively predicted support for racial equity policies, *B* = −0.42 [−0.48, −0.37], *p* < .001. No effect via attributions to ability was found, *ind* = 0.00 [−0.01, 0.01], *p* = .941.

#### External Attributions

Consistent with H_3_, our analysis revealed an indirect effect of Black poverty via our first external attribution measure, *ind* = −0.05 [−0.09, −0.01], *p* = .014. Black poverty negatively predicted attribution of racial inequality to a lack of educational opportunity, which *positively* predicted support for racial equity policies, *B* = 0.45 [0.40, 0.50], *p* < .001. No effect via attributions to discrimination was found, *ind* = −0.01 [−0.04, 0.03], *p* = .716.

#### Control Variables

Controlling for the same variables used in Study 1, the hypothesized total and indirect effects involving lack of motivation (internal attributions) remained significant, whereas those involving lack of educational opportunity (external attributions) became marginally significant (see Supplementary Materials, pp. 13–14).

## Study 3. Experiment. Black Poverty, Attributions of Racial Inequality, and Interracial Anxiety

Study 2 revealed that, among White Americans, county-level Black poverty predicted (a) stronger beliefs that racial inequality stems from a lack of motivation by Black Americans, (b) weaker beliefs that it stems from a lack of educational opportunity, and (c) indirect effects on support for racial equity policies. As Studies 1 and 2 used observational designs, Study 3 used an experimental approach to test the causal effects of Black poverty on attributions while examining the mediating role of interracial anxiety and identity threat. The study was preregistered (https://osf.io/entdm).^3^

### Method

#### Sample

A preregistered power analysis revealed that 1,034 participants were needed to detect mediation with small-sized paths of η^2^_p_s = .01 with *power* = .80 (see Supplementary Materials, p. 15). We recruited 1,040 White Americans using CloudResearch, with two interlocked quotas to match U.S. demographics for gender and education. After excluding four participants incorrectly identified as White by CloudResearch, the sample comprised 1,036 participants from 48 U.S. states and Washington, DC (see Supplementary Table S1 for sample characteristics).

#### Procedure and Variables

##### Induction of Black Poverty (Predictor)

Participants were asked to imagine visiting a place in the United States (a county, city, town, or neighborhood) and were randomly assigned to either the control condition (*n* = 507) or the experimental condition (*n* = 532). Participants in the control condition were asked to “imagine visiting a place in the U.S. where many Black residents live” and read:

The important thing is that the Black residents of this place appear to be similar to those in most regions of the US. You might come to realize this based on the way they look, speak, or behave. In any case, imagine visiting a place where you can tell that the Black residents are representative of the Black community in the US.

Participants in the experimental condition were asked to “imagine visiting a place in the U.S. where many of the Black residents earn little income” and read:

The important thing is that the Black residents of this place appear to be poor. You might come to realize this based on the way they look, speak, or behave. In any case, imagine visiting a place where you can tell that many of the Black residents are below the poverty line.

##### Variables

Following the induction, participants completed three measures (all response scales ranged from 1 = *Strongly disagree* to 7 = *Strongly agree*).

##### Interracial Anxiety (Mediator #1)

We adapted Stephan and Stephan’s (1985) measure, asking participants to rate how “tense,” “uneasy,” “bothered,” and “nervous” they believed they would feel when visiting the place (α = .97, *M* = 4.01, *SD* = 1.97).

##### Identity Threat (Mediator #2)

We adapted Bai et al.’s (2020) Symbolic Threat measure, asking participants to rate their agreement with three statements such as “The moral values of the Black residents of this place are not compatible with mine” and “Black residents in this place are undermining American cultural values” (α = .93, *M* = 2.71, *SD* = 1.72).

##### Attributions of Racial Inequality

We adapted Cozzarelli et al.’s (2001) Attributions for Poverty Scale, asking participants why “the Black residents of the place you have just imagined likely have on average worse jobs, income, and housing than White residents.” Three items measured *internal* attributions (e.g., “lack of effort,”α = .92, *M* = 3.28, *SD* = 1.77), and three measured *external* attributions (e.g., “denied an equal chance for education,”α = .91, *M* = 4.66, *SD* = 1.81). The subscales were negatively correlated, *r* = −0.42, *p* < .001.

### Results

#### Overview of the Structural Equation Model

**Figure 5:**
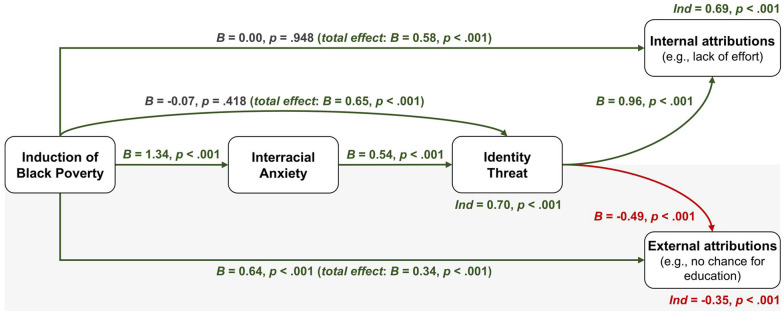
Study 3, H_4_—Paths of Interest: Effects of the Induction of Black Poverty on Internal Attributions (Upper Part) and External Attributions (Lower Part) of Racial Inequality, as Mediated by Interracial Anxiety and Identity Threat Among White Participants *Note.* Thick green lines (significantly positive *B*s) represent the positive effects; thick red lines (significantly negative *B*s) represent the negative effects; dashed gray lines (nonsignificant *B*s) represent the null effects. Latent variables were used; however, for the sake of readability, we do not display the individual items or their loading factors. “*ind*” stands for indirect effect, namely, *B*_poverty→anxiety_×*B*_anxiety→threat_ for the path reported below the “Identity Threat” box, and *B*_poverty→anxiety_×*B*_anxiety→threat_×*B*_threat→attributions_ for the other two.

The mediators were found to operate sequentially rather than concurrently, with the effect of identity threat reduced to null when interracial anxiety was included. As preregistered, we therefore built a structural equation model (SEM) to test the sequential influence of Black poverty on interracial anxiety, identity threat, and attributions. We included fixed effects to account for state-level clustering, and we calculated indirect effects using the percentile bootstrap method with 10^4^ resamples (Yzerbyt et al., 2018). We first report total effects (H_2a_-H_2b_) followed by mediational effects (*H*_4_). Figure 5 presents the overall model. The regression equations are presented in Supplementary Materials, p. 16.

#### H_2a-b_. Black Poverty → Internal/External Attributions of Racial Inequality (Total Effect)

Consistent with H_2a_, participants in the experimental condition used more internal attributions to explain racial inequality than those in the control condition, *B* = 0.58 [0.35, 0.80], *p* < .001. Inconsistent with H_2b_, they also used more, rather than less, external attributions, although the estimate appeared smaller, *B* = 0.34 [0.14, 0.55], *p* < .001.

#### H_4_. Black Poverty ^**+**^**→** Interracial Anxiety ^**+**^**→** Identity Threat → Attributions (Mediations)

Consistent with H_4a_, participants in the experimental condition experienced more identity threat than those in the control condition, *B* = 0.65 [0.46, 0.85], *p* < .001. This effect was mediated by interracial anxiety: Black poverty increased interracial anxiety, *B* = 1.34 [1.12, 1.57], *p* < .001, which positively predicted identity threat, *B* = 0.54 [0.49, 0.58], *p* < .001, with an indirect effect of *ind* = 0.70 [0.49, 0.92], *p* < .001.

Consistent with of H_4a_, identity threat was positively associated with internal attributions, *B* = 0.96 [0.90, 1.01], *p* < .001 and negatively associated with external attributions, *B* = −0.49 [−0.56, −0.42], *p* < .001. The total effect of Black poverty on internal attributions was mediated by interracial anxiety and identity threat, with a positive indirect effect of *ind* = 0.69 [0.56, 0.83], *p* < .001. However, the inconsistent total effect of Black poverty on external attributions remained unchanged when interracial anxiety and identity threat were included in the model, with a negative indirect effect of *ind* = −0.36 [−0.44, −0.28], *p* < .001. Controlling for the same participant-level variables used in Studies 1 and 2, the conclusion from the main analysis remained the same (for the results, see Supplementary Figure S1).

## Discussion

In this research, we argued that Black poverty threatens White Americans, fostering the self-protective belief that Black Americans are responsible for their own hardship. Below, we discuss this phenomenon and then the underlying processes.

### Exposure to Black Poverty and Attribution of Racial Inequality

Study 1 demonstrated that county-level Black poverty rates predicted stronger beliefs in racial equality of opportunity among White Americans, but weaker beliefs among Black Americans. Interestingly, Hispanic Americans exhibited a response similar to that of White Americans, aligning with research showing that the attribution beliefs of these two groups have converged in recent decades (Hunt, 2007). This may reflect the fact that most Hispanic Americans—over 75% according to the GSS data^4^—identify as White (Bernstein, 2022) and have become more conservative over time (Fraga et al., 2024).

In Studies 2 and 3, we observed that Black poverty led White Americans to attribute racial inequality to internal factors. Importantly, in Study 2, White Americans favored internal explanations rooted in differences in motivation (effort-based) rather than innate capability (ability-based). These attributions, in turn, predicted decreased support for racial equity policies. This aligns with evidence that effort-based attributions have replaced ability-based attributions as the dominant form of internal attributions (Hunt, 2007), offering a more socially acceptable way to express anti-Black sentiment (Kluegel, 1990) and forming a core component of modern racism (Gomez & Wilson, 2006).

However, the relationship between Black poverty and external attributions proved less consistent. In Study 2, Black poverty was associated with White Americans downplaying differences in educational opportunity as a cause of racial inequality (an effect that became marginally significant with controls) but did not affect their views on discrimination. This discrepancy may stem from the fact that equality of opportunity is a core American value (Davidai, 2022), whereas denying discrimination is socially undesirable (Sanchez et al., 2024). Adding to the complexity, in Study 3, Black poverty led White Americans to make more, not fewer, external attributions. This may be due to the strength of the induction, which may have prompted participants to consider a broader range of explanations for Black poverty. Overall, while Black poverty consistently predicts internal attributions, findings for external attributions were mixed.

### Interracial Anxiety and Identity Threat as Psychological Mechanisms

Study 3 demonstrated that the effect of Black poverty on internal attributions was mediated by interracial anxiety and identity threat. Specifically, exposure to Black poverty increased interracial anxiety, which then predicted identity threat and, ultimately, internal attributions. These results suggest that Black poverty may provoke an emotional response among White Americans, fostering concerns about the outgroup threatening American identity and values. Putting the blame for poverty on Black Americans themselves may serve as a psychological mechanism to deflect negative emotions, maintain a positive group identity, and mitigate feelings of responsibility for racial inequality (Branscombe et al., 2007; Ford et al., 2022; Wohl et al., 2006). However, Study 3 also documented an unexpected positive effect on external attributions. While identity threat was negatively associated with external attributions, the mechanisms behind such an effect appear more intricate.

Our findings align with research suggesting that prejudice results from justifications of inequality meant to protect one’s feelings and identity (Fein & Spencer, 1997; Sherman et al., 2017; Tajfel & Turner, 1979). Social Identity Theory has traditionally focused on how identity-related threats and affirmations influence prejudice. Here, we examined an implication of the theory that has received much less attention: how spatial variation in racial inequality itself can provoke different levels of justification (Acharya et al., 2016; Payne et al., 2019). Research on the “bias of crowds” suggests that U.S. regions where Black Americans have historically been disadvantaged often display elevated levels of implicit bias (Payne et al., 2019; Riddle & Sinclair, 2019; Vuletich et al., 2024). These aggregated biases are seen as local markers of structural racism (Payne et al., 2017), and our findings suggest that White Americans in these areas may feel particularly motivated to rationalize existing inequality.

### Limitations and Conclusion

One limitation of this research is that Study 3 used a measurement-of-mediation design. Interpreting mediation with measured variables requires caution (Pósch, 2021), particularly due to issues such as measurement error (Valeri & Vanderweele, 2014). Future studies using experimental mediation are needed (Pirlott & MacKinnon, 2016). Another limitation is that the hypothesized effects were sometimes affected by the inclusion of demographic control variables. In Study 1, the interaction between Black poverty and race was unaffected, but the simple effect among White participants became nonsignificant. In Study 2, the effects on internal attributions were unaffected, but those on external attributions became marginal. In Study 3, the effects on both types of attributions were unaffected. Future research should examine processes related to socioeconomic status or political orientation. A final limitation is the focus on a specific group and context (race/ethnicity in the United States). Since internal attributions can alleviate the discomfort of being viewed as unfairly privileged relative to any group (Leach et al., 2002), our results may extend to other marginalized groups. Future research could examine whether poverty affecting other minority groups (ethnic, religious, or cultural) prompts majority group members to blame inequality on the minority itself.

## Supplemental Material

sj-docx-1-spp-10.1177_19485506251329122 – Supplemental material for Black Poverty Leads White Americans to Blame Racial Inequality on Black Americans Themselves Supplemental material, sj-docx-1-spp-10.1177_19485506251329122 for Black Poverty Leads White Americans to Blame Racial Inequality on Black Americans Themselves by Nicolas Sommet and B. Keith Payne in Social Psychological and Personality Science

## Notes

1. In this and the subsequent study, we repeated the main analysis using an alternative predictor of Black poverty. The analyses, which were overall inconclusive across the two studies, are presented in the Supplementary Materials, pp. 5–6 (Study 1) and pp. 11–12 (Study 2).

2. We preregistered an additional analysis testing the effect of county-mean-centered Black poverty. However, the mean year-to-year correlation was .75, indicating minimal change in Black poverty over time. Hence, we focused on between-county associations and addressed causality in a third, experimental study.

3. We initially conducted another preregistered experiment testing a similar model but replaced it with the current experiment because of methodological issues identified during the revision process (this experiment is reported in Supplementary Materials).

4. For the script of the analysis, see the OSF.
