# Supplementary material for: Black Poverty Leads White Americans to Blame Racial Inequality on Black Americans Themselves
Source: Soc Psychol Personal Sci. 2025 Apr 3;17(2):229–41. doi: 10.1177/19485506251329122 (PMC12795330; doi:10.1177/19485506251329122)
Supplement: sj-docx-1-spp-10.1177_19485506251329122 – Supplemental material for Black Poverty Leads White Americans to Blame Racial Inequality on Black Americans Themselves [file sj-docx-1-spp-10.1177_19485506251329122.docx]

**Supplementary Materials (SM) for**

**Contexts of Black Poverty Lead White Americans to Blame
Racial Inequality on Black Americans Themselves**

This document is organized as follow:

• P. 2. **Table S1. Studies 1-3:** Sample characteristics

**Study 1.**

• P. 3. **Study 1:** Sensitivity analysis

• P. 4. **Study 1:** Multilevel Regression Equation

• P. 5. Supplementary Analysis Using the Alternative Black Poverty Indicator

• P. 6. **Table S2.** **Study 1:** Alternative Black Poverty Indicator [full results]

• P. 7. Repeated the Analysis With the Control Variables

• P. 8. **Table S3.** **Study 1:** Analysis with the Control Variables [full results]

**Study 2.**

• P. 9. **Study 2:** Sensitivity analysis

• P. 10. **Study 2:** Multilevel Regression Equation

• P. 11. Supplementary Analysis Using the Alternative Black Poverty Indicator

• P. 12. **Table S3. Study 2:** Alternative Black Poverty Indicator

• P. 13. Repeated the Analysis with the Control Variables

• P. 14. **Table S4.** **Study 2:** Repeating the Analysis with the Control Variables

**Study 3.**

• P. 15. **Study 3:** Power analysis

• P. 16. **Study 3:** Structural Equation Model

• P. 17. **Figure S1, Study 3:** Repeating the Analysis with the Control Variables

**Former Study 3.**

• P. 18. Report of former Study 3 (replaced by current Study 3 during the revision process)

# Table S1. Studies 1-3: Sample Characteristics

*Participant-level and country-level characteristics*

|  | **Study 1.**  **U.S. Gallup** | **Study 2.**  **GSS** | **Study 3.**  **Experiment** |
| --- | --- | --- | --- |
| **Participant-level characteristics** |  |  |  |
| Proportion of men | .49 [.48, .50] | .47 [.45, .48] | .49 [.47, .53] |
| Mean age^a^ | 51.3 [51.0, 51.7] | 48.0 [47.5, 48.6] | 43.7 [42.8, 44.5] |
| Proportion of married participants | .47 [.46, .49] | .47 [.46, .49] | .53 [.50, .56] |
| Proportion in the workforce | .36 [.35, .37] | .61 [.60, .63] | .72 [.69, .75] |
| Education^a,b^ | .43 [.43, .44] | 13.9 [13.8, 14.0] | .35 [.32, .38] |
| Mean annual equalized income (in US$ 10^4^) | 9.23 [9.01 9.45] | 10.7 [10.5, 10.9] | 8.74 [8.41, 9.08] |
| Mean political orientation^c^ | 3.54 [3.51, 3.57] | 2.90 [2.84, 2.96] | 3.53 [3.41, 3.64] |
| **County-level characteristics** |  |  |  |
| Mean population (in 10^5^ inhabitants) | 5.42 [5.05, 5.79] | 9.29 [8.51, 10.1] | n/a |
| Mean proportion of Black v. White residents | .21 [.21, .22] | .20 [.19, .21] | n/a |
| Mean poverty rate among White residents | .10 [.09, .10] | .09 [.08, .09] | n/a |
| Median annual household income  (in 10^4^ inflation-adjusted USD) | 6.26 [6.18, 6.35] | 5.96 [5.85, 6.07] | n/a |

*Note*. To determine income from the closed-ended household income items, we took the midpoint of the income band (for all but the last categories), or derived the value from Hout’s (2004) Pareto curve-based formula (for the last category); to adjust for household size, we used OECD’s (2009) equivalization formula (i.e., $\text{income}_{\text{adj}}\text{= }\text{income}\text{ ÷ }\sqrt{\text{household size}}$); “n/a” means “not applicable.”

^a^ Study 3: We used two interlocked quotas to create a representative sample in terms of gender (≈½ men; ≈½ women) and education (≈40% high school or less; ≈25% some college/associate degree; ≈35% 4-year college degree or more; U.S. Census Bureau, 2022).

^b^ Studies 1-3: Proportion of college-graduated participants; Study 2: Mean years of education

^c^ Study 1: 1 = *Republican* to 5 = *Democrat*; Study 2: 0 = *Strong democrat* to 6 = *Strong republican*; Study 3: 1 = *Very liberal* to 5 = *Very conservative*

# Study 1: Simulation-Based Sensitivity Analyses

We used the R package *simglm* (LeBeau, 2019) to simulate 10,000 three-level datasets with the same number of level-1, level-2, and level-3 units as in the actual sample. For each dataset, we simulated (i) a level-1 outcome “*opp*_ijk_,” drawn from a binomial distribution, (ii) a level-2 categorical predictor “*race*_jk_,” using the same probability for each group to be sampled as in the actual sample (i.e, 50%, 28%, and 22% for *race*_jk_ = White, Black, and Hispanic, respectively), and (iii) a level-3 continuous predictor “*Black poverty*_k_,” drawn from a normal distribution with μ = 0, σ = 1. We ran the same multilevel logistic model used in the main analysis (see Eq. 1) for each of the 10,000 simulated datasets. Regarding the population random parameters, we used the same level-2 and level-3 residual variance (i.e., random intercept variance) as in the actual sample, and—for the sake of computational feasibility—we set the slope residual variance (i.e., random slope variance) and covariance terms to zero. Regarding the population fixed parameters, we used β = 0.18 (i.e., OR = exp(0.18) = 1.20) for the critical “$\text{d1}_{\text{race}_{\text{jk}}}\text{ }\text{×}\text{ }\text{Black poverty}_{\text{k}}$” effect, whereas all the other coefficients were set to zero. This corresponded to a small-sized fully attenuated interaction effect (Blake & Gangestad, 2020; Sommet et al., 2023) with (i) a positive simple effect of Black poverty of OR = 1.44 for White participants (i.e., *r* = .10) and (ii) a null simple effect of Black poverty of OR = 1.00 for Black participants (i.e., *r*s = .00). The simulation-based sensitivity analysis revealed that our sample was sufficient to detect such an interaction using a two-tailed test with α = .05 with a power of .96. The scripts of the sensitivity analyses can be found on the OSF page of the project.

# Study 1. Multilevel Regression Equation

Below is the equation of the regression model used to test H_1_:

|  | $\text{logit}\left( \text{P}\left( \text{opp}_{\text{ijk}}\text{ = 1} \right) \right)\text{= }\text{β}_{\text{000}}\text{ + }\text{β}_{\text{001}}\text{ × }\text{Black poverty}_{\text{k}}\text{ + }\left( \text{β}_{\text{010}}\text{ }{\text{+ }\text{v}}_{\text{01k}} \right)\text{ × }\text{d1}_{\text{race}_{\text{jk}}}\text{ + }\left( \text{β}_{\text{020}}\text{ }{\text{+ }\text{v}}_{\text{02k}} \right)\text{ × }$  ${\text{d2}_{\text{race}_{\text{jk}}}\text{ }\text{+}\text{ }\text{β}}_{\text{011}}\text{ }\text{×}\text{ }\text{d1}_{\text{race}_{\text{jk}}}\text{ }\text{×}\text{ }\text{Black poverty}_{\text{k}}\text{ }\text{+}\text{ }\text{β}_{\text{02}\text{1}}\text{ }\text{×}\text{ }\text{Black poverty}_{\text{k}}\text{ }\text{×}\text{ }\text{d2}_{\text{race}_{\text{jk}}}\text{ }{\text{+ }\text{δ}}_{\text{t}\text{-1}}\text{ }{\text{+ }\text{u}}_{\text{0jk}}{\text{ }\text{+ }\text{v}}_{\text{00k}}$ | (1) |
| --- | --- | --- |

…*i* = 1, 2, …, *N* [within-participant responses], *j* = 1, 2, …, *K* [participants], *k* = 1, 2, …, *L* [counties], where *opp*_ijk_ represents beliefs in racial equality of opportunity, *Black poverty*_k_ represents county-level Black poverty, $\text{d1}_{\text{race}_{\text{jk}}}$ represents the first race/ethnicity-related dummy (comparing White and Black participants) and $\text{d2}_{\text{race}_{\text{jk}}}$ represents the second race/ethnicity-related dummy (comparing White and Hispanic participants), and where δ_t-1_ represents the year fixed-effects (*t* = 1, 2, …, 5 [years]), *v*_01k_/*v*_02k_ are the county-level slope residuals (i.e., random slope), *u*_0jk_ is the level-2 error term, and *v*_00k_ is the level-3 error term.

# Study 1. Supplementary Analysis Using the Alternative Black Poverty Indicator

We conducted a supplementary analysis using an alternative Black Poverty indicator. Instead of using the county-level poverty rate among Black residents (i.e., the extent to which Black people are below the poverty line), we used the county-level proportion of Black individuals among those living below the poverty line (i.e., the extent to which people living below the poverty line are Black). This measure ranged from .004 (less than 1% of people living below the poverty line are Black) to .88 (88% of people living below the poverty line are Black) (*M* = .29, *SD* = .19).

We ran the same model used in the main analysis (described on the previous page), substituting the Black poverty indicator from the main analysis with this alternative measure. The interaction between the alternative Black poverty variable and race/ethnicity was marginally significant, χ^2^ (2, *N* = 11,885) = 4.80, *p* = .09. However, as shown in **Table S2** on the next page, the interaction between Black poverty and the focal Black vs. White dummy variables remained significant (see *B*_011_), though the simple effect of Black poverty among Black residents was not significant (see *B*_001_). To gain greater clarity, we preregistered an analysis using this indicator in Study 2.

# Table S2. Study 1: Alternative Operationalization

*ORs/95% CIs from the model testing the association between Black poverty and beliefs in racial equality of opportunity as a function of race/ethnicity while using the alternative operationalization of Black poverty.*

|  |  | *OR* | 95% |
| --- | --- | --- | --- |
| Focal variables | Intercept, *B*_000_ | 3.89^***^ | [3.46, 4.38] |
|  | County-level Black poverty (standardized), *B*_001_ | 1.04 | [0.96, 1.12] |
|  | Race/Ethnicity: Dummy #1 (White [baseline] vs. Black), *B*_010_ | 0.18^***^ | [0.17, 0.20] |
|  | Dummy #2 (White [baseline] vs. Hispanic), *B*_020_ | 0.67^***^ | [0.59, 0.76] |
|  | Interaction^A^: Black poverty × Dummy #1, *B*_011_ | 0.91^*^ | [0.83, 0.99] |
|  | Black poverty × Dummy #2, *B*_021_ | 0.99 | [0.87, 1.13] |
| Fixed effects | Year 2007 (baseline) vs. 2008, δ_1_ | 1.14 | [1.00, 1.31] |
|  | 2013, δ_2_ | 1.19^**^ | [1.06, 1.34] |
|  | 2015, δ_3_ | 1.02 | [0.90, 1.17] |
|  | 2018, δ_4_ | 0.23^***^ | [0.20, 0.25] |
| Variance parameters | Level-2 residual variance (random intercept), var(*u*_0jk_) | 1.68 |  |
|  | Level-3 residual variance (random intercept), var(*v*_00k_) | 0.22 |  |
|  | Level-3 residual slope variance (random slope) Dummy #1, var(*v*_01k_) | 0.08 |  |
|  | Level-3 residual slope variance (random slope) Dummy #2, var(*v*_02k_) | 0.15 |  |
|  | Covariance parameter #1, cov(*u*_0jk_, *v*_01k_) | -0.12 |  |
|  | Covariance parameter #2, cov(*u*_0jk_, *v*_02k_) | -0.10 |  |

*Note*. The coefficient estimate corresponding to the hypothesis is highlighted in yellow.

^A^ The omnibus interaction between Black poverty and race/ethnicity (White vs. Black vs. Hispanic American) is marginally significant,χ^2^ (2, *N* = 11,885) = 4.80, *p* = .09.

^***^*p* < .001, ^**^*p* < .01, ^*^*p* < .05

# Study 1. Supplementary Analysis With the Control Variables

We repeated the main analysis, this time controlling for an a priori-defined set of seven participant-level demographics (sex, age, marital status, working status, education, log-transformed equivalized household income, and political party identification) and four county-level socioeconomic variables (population, Black vs. White resident ratio, White poverty, and log-transformed median income in the area).

The Black poverty × race/ethnicity interaction remained significant, χ^2^ (2, *K* = 10,666) = 18.70, *p* < .001. As shown in **Table S3** on the next page, the interaction between Black poverty and the focal Black vs. White dummy variables also remained significant (see *B*_011_). However, the simple effect of Black poverty among White residents was not significant (see *B*_001_). These results suggest that the observed finding—that White individuals in areas with higher levels of Black poverty are more likely to endorse beliefs in racial equality—might be explained by underlying socioeconomic and political differences. This issue will be further examined in Study 3, which investigates the causal effect of Black poverty through an experimental approach.

# Table S3. Study 1: Control Variables

*ORs/95% CIs from the model testing the association between Black poverty and beliefs in racial equality of opportunity as a function of race/ethnicity while including control variables*

|  |  | *OR* | 95% CI |
| --- | --- | --- | --- |
| Focal variables | Intercept, *B*_000_ | 37.31^***^ | [20.5, 68.0] |
|  | County-level Black poverty (standardized), *B*_001_ | 0.99 | [0.92, 1.06] |
|  | Race/Ethnicity: Dummy #1 (White [baseline] vs. Black), *B*_010_ | 0.30^***^ | [0.27, 0.33] |
|  | Dummy #2 (White [baseline] vs. Hispanic), *B*_020_ | 0.78^***^ | [0.69, 0.89] |
|  | Interaction^A^: Black poverty × Dummy #1, *B*_011_ | 0.83^***^ | [0.74, 0.92] |
|  | Black poverty × Dummy #2, *B*_021_ | 1.10 | [0.97, 1.26] |
| County- and participant-level control variables | Gender (0 = *women*; 1 = *men*), *B*_030_ | 0.98 | [0.91, 1.06] |
|  | Age (1 unit = 10 years), *B*_040_ | 1.01 | [0.99, 1.04] |
|  | Marital status (0 = *not married*; 1 = *married*), *B*_050_ | 1.16^***^ | [1.07, 1.25] |
|  | Employment status (0 = *not working*; 1 = *working*), *B*_060_ | 1.06 | [0.97, 1.17] |
|  | Education (0 = *not graduated*; 1 = *college-graduated*), *B*_070_ | 0.58^***^ | [0.54, 0.63] |
|  | Log-transformed equalized income (1 unit = USD 10^4^), *B*_080_ | 0.97^*^ | [0.95, 0.99] |
|  | Political orientation, *B*_090_ | 0.68^***^ | [0.66, 0.69] |
|  | Population (1 unit = 10^5^ inhabitants), *B*_002_ | 0.996^*^ | [0.99, 1.00[ |
|  | Proportion of Black residents, *B*_003_ | 0.96^*^ | [0.91, 1.00[ |
|  | White Poverty, *B*_004_ | 0.95 | [0.88, 1.02] |
|  | Log median annual household income (in real USD 10^4^), *B*_005_ | 0.60^**^ | [0.45, 0.81] |
| Fixed effects | Year 2007 (baseline) vs. 2008, δ_1_ | 1.16^**^ | [1.01, 1.33] |
|  | 2013, δ_2_ | 1.10^*^ | [0.96, 1.26] |
|  | 2015, δ_3_ | 0.92 | [0.80, 1.07] |
|  | 2018, δ_4_ | 0.22^***^ | [0.19, 0.25] |
| Variance parameters | Level-2 residual variance (random intercept), var(*u*_0jk_) | 1.38 |  |
|  | Level-3 residual variance (random intercept), var(*v*_00k_) | 0.03 |  |
|  | Level-3 residual slope variance (random slope) Dummy #1, var(*v*_01k_) | 0.00 |  |
|  | Level-3 residual slope variance (random slope) Dummy #2, var(*v*_02k_) | 0.15 |  |
|  | Covariance parameter #1, cov(*u*_0jk_, *v*_01k_) | 0.00 |  |
|  | Covariance parameter #2, cov(*u*_0jk_, *v*_02k_) | -0.02 |  |

*Note*. The coefficient estimate corresponding to the hypothesis is highlighted in yellow.

^A^ The omnibus interaction between Black poverty and race/ethnicity (White vs. Black vs. Hispanic American) is marginally significant,χ^2^ (2, *N* = 11,885) = 4.80, *p* = .09.

^***^*p* < .001, ^**^*p* < .01, ^*^*p* < .05

# Study 2: Simulation-Based Sensitivity Analyses

We again used the R package *simglm* to simulate 10,000 two-level datasets with the same number of level-1 and level-2 units as in the actual sample. For each dataset, we simulated (i) a level-1 outcome “$\text{att}_{\text{n}_{\text{ij}}}$,” drawn from a binomial distribution, and (ii) a level-2 continuous predictor “*Black poverty*_k_,” drawn from a normal distribution with μ = 0, σ = 1. We ran the same multilevel logistic model used in the main analysis (see Eq. 2, p. 5). Regarding the population random parameters, we used the same level-2 residual variance (i.e., random intercept variance) as the mean variance for the four outcome variables used in the actual sample. Regarding the population fixed parameters, we used β = 0.36 (i.e., OR = exp(0.36) = 1.44) for the critical “$\text{Black poverty}_{\text{k}}$” effect. This corresponded to a small-sized main effect of Black poverty (Gignac & Szodorai, 2016). The simulation-based sensitivity analysis revealed that our sample was sufficient to detect such a main effect using a two-tailed test with α = .05 with a power of .9999, meaning that our sample was sufficient to detect *four* of such interactions with a power of .9999^4^ = .9996. The scripts of the sensitivity analyses can be found on the OSF page of the project.

# Study 2. Multilevel Regression Equations

Below is the equation of the regression model used to test H_2a-b_:

|  | $\text{logit}\left( \text{P}\left( \text{att}_{\text{n}_{\text{ij}}}\text{ = 1} \right) \right)\text{= }\text{β}_{\text{00}}\text{ + }\text{β}_{\text{01}}\text{ × }\text{Black poverty}_{\text{j}}\text{ +}{\text{ }\text{δ}}_{\text{t-1}}\text{ }{\text{+ }\text{u}}_{\text{0j}}$ | (2) |
| --- | --- | --- |

…*i* = 1, 2, …, *N* [participants], *j* = 1, 2, …, *K* [counties], where $\text{att}_{\text{n}_{\text{ij}}}$represents the attribution scores and *Black poverty*_j_ represents county-level Black poverty, and where δ_t-1_ represents the year fixed-effects (*t* = 1, 2, …, 6 [years]), and *u*_0j_ is the level-2 error term.

Below is the equation of the regression model used to test H_3_:

|  | $\text{supp}_{\text{ij}}\text{ = }\text{β}_{\text{00}}\text{ + }\text{β}_{\text{01}}\text{ × }\text{Black poverty}_{\text{j}}\text{ + }\left\{ \text{β}_{\text{10}}\text{ × }\text{att}_{\text{1}_{\text{ij}}}\text{ + }\text{β}_{\text{20}}\text{ × }\text{att}_{\text{2}_{\text{ij}}}\text{ + }\text{β}_{\text{30}}\text{ × }\text{att}_{\text{3}_{\text{ij}}}\text{ + }\text{β}_{\text{40}}\text{ × }\text{att}_{\text{4}_{\text{ij}}} \right\}\text{ + }{\text{ }\text{δ}}_{\text{t-1}}\text{ }{\text{+ }\text{u}}_{\text{0j}}\text{ }{\text{+ }\text{e}}_{\text{i}\text{j}}$ | (3) |
| --- | --- | --- |

…*i* = 1, 2, …, *N* [participants], *j* = 1, 2, …, *K* [counties], where *supp*_ij_ represents support for racial equity policies, $\text{att}_{\text{n}_{\text{ij}}}$represents the attribution scores, and *Black poverty*_j_ represents county-level Black poverty, and where the regressors for the second stage of the analysis are shows in curly brackets, δ_t-1_ represents the year fixed-effects (*t* = 1, 2, …, 6 [years]), and *u*_0j_ and *e*_ij_ are the level-2 and level-1 error terms, respectively.

# Study 2. Supplementary Analysis Using the Alternative Black Poverty Indicator

As in Study 1, we conducted a supplementary analysis using the county-level proportion of Black individuals among poor residents as an alternative Black Poverty indicator. The measure ranged from .01 (1% of people living below the poverty line are Black) to .83 (83% of people living below the poverty line are Black) (*M* = .25, *SD* = .17).

We ran the same model used in the main analysis (described on the previous page), substituting the Black poverty indicator from the main analysis with this alternative measure. As shown in **Table S4**, the alternative Black poverty indicator did not predict any of the four outcomes, *p*s ≥ .376. These findings suggests that the socioeconomic profiles of the Black population in one’s local environment may be more noticeable than the racial composition of the population living below the poverty line, and may therefore better predict attributions of racial inequality.

# Table S4. Study 2: Alternative Operationalization

*ORs/Bs and 95% CIs from the models testing the association between Black poverty and attributions and the downstream statistical effects on support for racial equity policies while using the alternative operationalization*

|  |  | **Black Poverty to Attributions of Racial Inequality (H_2a-b_)** | | | | | | | | **Downstream effects (H_3_)** | | | |
| --- | --- | --- | --- | --- | --- | --- | --- | --- | --- | --- | --- | --- | --- |
|  |  | Internal attributions | | | | External attributions | | | | Support for Racial Equity Policies | | | |
|  |  | Lack of motivation (*att*_1_) | | Less in-born ability (*att*_2_) | | No chance for education (*att*_3_) | | Discrimination (*att*_4_) | | Total effect | | Mediator and direct effects | |
|  |  | *OR* | 95% CI | *OR* | 95% CI | *OR* | 95% CI | *OR* | 95% CI | *B* | 95% CI | *B* | 95% CI |
| Focal variables | Black poverty (std), *B*_01_ | 1.01 | [0.92, 1.10] | 1.06 | [0.94, 1.19] | 1.01 | [0.92, 1.11] | 0.98 | [0.90, 1.07] | 0.01 | [-0.02, 0.04] | 0.02 | [-0.01, 0.05] |
|  | Lack of motivation, *B*_10_ |  |  |  |  |  |  |  |  |  |  | -0.42^***^ | [-0.47, -0.37] |
|  | Less in-born ability, *B*_20_ |  |  |  |  |  |  |  |  |  |  | -0.07 | [-0.16, 0.03] |
|  | No chance for education, *B*_30_ |  |  |  |  |  |  |  |  |  |  | 0.45^***^ | [0.39, 0.50] |
|  | Discrimination, *B*_40_ |  |  |  |  |  |  |  |  |  |  | 0.62^***^ | [0.56, 0.67] |
| Year fixed-effects | 2006 (baseline) vs. 2008, δ_1_ | 1.19 | [0.93, 1.50] | 1.27 | [0.87, 1.85] | 1.22 | [0.96, 1.56] | 1.15 | [0.90, 1.49] | 0.07 | [-0.04, 0.18] | 0.04 | [-0.06, 0.13] |
|  | 2010, δ_2_ | 0.86 | [0.70, 1.06] | 0.99 | [0.69, 1.41] | 1.17 | [0.94, 1.44] | 1.08 | [0.86, 1.35] | 0.04 | [-0.06, 0.14] | 0 | [-0.08, 0.08] |
|  | 2012, δ_3_ | 0.89 | [0.72, 1.12] | 0.77 | [0.52, 1.13] | 0.93 | [0.74, 1.16] | 1.09 | [0.86, 1.38] | 0.03 | [-0.07, 0.13] | 0.01 | [-0.07, 0.10] |
|  | 2014, δ_3_ | 0.75^**^ | [0.61, 0.92] | 0.77 | [0.54, 1.11] | 0.97 | [0.78, 1.19] | 0.95 | [0.76, 1.19] | 0.09 | [-0.00, 0.18] | 0.07 | [-0.01, 0.15] |
|  | 2016, δ_4_ | 0.68^***^ | [0.56, 0.84] | 0.76 | [0.53, 1.08] | 1.46^***^ | [1.19, 1.80] | 1.60^***^ | [1.30, 1.97] | 0.34^***^ | [0.25, 0.43] | 0.20^***^ | [0.12, 0.28] |
| VP | L2 residual variance, var(*u*_0j_) | 0.16 |  | 0.04 |  | 0.21 |  | 0.12 |  | 0.95 |  | 0.69 |  |

*Note*: The coefficient estimates corresponding to the hypotheses are highlighted in yellow; “VP” = Variance parameters, “std” = standardized.

^***^*p* < .001, ^**^*p* < .01, ^*^*p* < .05

**Study** **2: Supplementary Analysis With the Control Variables**

We repeated the main analysis, controlling for the same variables used in Study 1.This time, preliminary analyses indicated collinearity issues when including county-level median income. Specifically, the correlation between county-level Black poverty and median income was *r* = -0.63, *p* < .001, and the VIF was 3.67 (VIF > 2.5 is a cause for concerns in logistic regression; Midi et al., 2010). Deviating from the preregistration, we therefore exclude county-level median income from the set of control variables

As how in **Table S5**, the associations observed in the main analysis remained roughly the same when controlling for our set of control variables. (i) Black poverty positively predicted attribution of racial inequality to a lack of motivation, *B* = 0.11 [0.01, 0.21], *p* = .028 (H_2a_), which itself negatively predicted support for racial equity policies, *B* = -0.34 [-0.40, -0.29], *p* < .001; *ind* = -0.04 [-0.07, -0.003], *p* = .029 (H_3_); (ii) Black poverty had a marginally significant negative effect on attributing racial inequality to a lack of educational opportunity, *B* = -0.08 [-0.18, 0.01], *p* = .088 (H_2a_), which itself positively predicted support for racial equity policies, *B* = 0.38 [0.32, 0.44], *p* < .001; with a marginally significant indirect effect, *ind* = -0.03 [-0.07, 0.002], *p* = .066 (H_3_).

# Table S5. Study 2: Control Variables

*ORs/Bs and 95% CIs* *from the models testing the association between Black poverty and attributions and the downstream statistical effects on support for racial equity policies while including control variables*

|  |  | **Black Poverty to Attributions of Racial Inequality (****H_2a-b_)** | | | | | | | | | **Downstream effects (H_3_)** | | | |
| --- | --- | --- | --- | --- | --- | --- | --- | --- | --- | --- | --- | --- | --- | --- |
|  |  | Internal attributions | | | | External attributions | | | | | Support for Racial Equity Policies | | | |
|  |  | Lack of motivation (*att*_1_) | | Less in-born ability (*att*_2_) | | No chance for education (*att*_3_) | | Discrimination (*att*_4_) | | | Total effect | | Mediator and direct effects | |
|  |  | *OR* | 95% CI | *OR* | 95% CI | *OR* | 95% CI | | *OR* | 95% CI | *B* | 95% CI | *B* | 95% CI |
| Focal vars | Black poverty (standardized), *B*_01_ | 1.11^*^ | [1.01, 1.23] | 1.01 | [0.86, 1.19] | 0.92^✝^ | [0.84, 1.01] | | 0.96 | [0.87, 1.05] | -0.02 | [-0.06, 0.02] | 0.00 | [-0.03, 0.03] |
|  | Lack of motivation, *B*_10_ |  |  |  |  |  |  | |  |  |  |  | -0.34^***^ | [-0.40, -0.29] |
|  | Less in-born ability, *B*_20_ |  |  |  |  |  |  | |  |  |  |  | -0.02 | [-0.12, 0.08] |
|  | No chance for education, *B*_30_ |  |  |  |  |  |  | |  |  |  |  | 0.38^***^ | [0.32, 0.44] |
|  | Discrimination, *B*_40_ |  |  |  |  |  |  | |  |  |  |  | 0.55^***^ | [0.49, 0.61] |
| County- & participant-level vars | Gender (1 = *men*), *B*_05_ | 1.10 | [0.96, 1.27] | 1.16 | [0.89, 1.50] | 0.99 | [0.86, 1.14] | | 0.89 | [0.77, 1.03] | 0.01 | [-0.05, 0.07] | 0.04 | [-0.02, 0.09] |
|  | Age (1 unit = 10 years), *B*_06_ | 1.07^**^ | [1.02, 1.13] | 1.17^***^ | [1.07, 1.28] | 1.02 | [0.97, 1.07] | | 1.03 | [0.98, 1.08] | -0.02 | [-0.04, 0.00] | -0.02^✝^ | [-0.04, 0.00] |
|  | Never vs. was married, *B*_07_ | 0.99 | [0.79, 1.24] | 1.03 | [0.69, 1.53] | 0.76^*^ | [0.61, 0.94] | | 0.85 | [0.67, 1.06] | -0.08^✝^ | [-0.17, 0.01] | -0.03 | [-0.11, 0.05] |
|  | is married, *B*_08_ | 1.04 | [0.86, 1.26] | 0.90 | [0.63, 1.29] | 0.74^**^ | [0.61, 0.89] | | 0.69^***^ | [0.57, 0.84] | -0.14^***^ | [-0.21, -0.06] | -0.06^✝^ | [-0.13, 0.01] |
|  | Status (1 = *working*), *B*_09_ | 0.87^✝^ | [0.74, 1.02] | 0.84 | [0.63, 1.13] | 0.89 | [0.76, 1.04] | | 0.75^***^ | [0.64, 0.89] | -0.01 | [-0.08, 0.06] | 0.02 | [-0.04, 0.08] |
|  | Years of education, *B*_010_ | 0.85^***^ | [0.83, 0.87] | 0.85^***^ | [0.82, 0.89] | 1.16^***^ | [1.13, 1.19] | | 1.02 | [1.00, 1.05] | 0.06^***^ | [0.04, 0.07] | 0.03^***^ | [0.02, 0.04] |
|  | Log Income (in USD 10^4^), *B*_011_ | 0.98 | [0.92, 1.04] | 0.89^*^ | [0.80, 0.97] | 0.98 | [0.92, 1.04] | | 0.93^*^ | [0.87, 0.98] | -0.05^***^ | [-0.07, -0.02] | -0.04^***^ | [-0.06, -0.02] |
|  | Political orientation, *B*_012_ | 1.21^***^ | [1.17, 1.26] | 1.03 | [0.97, 1.10] | 0.83^***^ | [0.80, 0.86] | | 0.77^***^ | [0.74, 0.80] | -0.16^***^ | [-0.17, -0.14] | -0.09^***^ | [-0.11, -0.08] |
|  | Population (1 unit = 10^5^), *B*_20_ | 1.00 | [0.99, 1.01] | 1.00 | [0.99, 1.01] | 1.00 | [1.00, 1.01] | | 1.00 | [1.00, 1.01] | 0.00^*^ | [0.00, 0.00] | 0.00 | [-0.00, 0.00] |
|  | Proportion Black residents, *B*_30_ | 1.00 | [0.91, 1.11] | 1.10 | [0.96, 1.27] | 0.99 | [0.90, 1.08] | | 1.06 | [0.97, 1.17] | 0.02 | [-0.02, 0.05] | 0.02 | [-0.01, 0.05] |
|  | White Poverty, *B*_40_ | 1.07 | [0.97, 1.18] | 0.96 | [0.82, 1.12] | 0.96 | [0.87, 1.05] | | 0.99 | [0.90, 1.10] | -0.02 | [-0.05, 0.02] | -0.01 | [-0.04, 0.02] |
| Fixed effects | 2006 (baseline) vs. 2008, δ_1_ | 1.17 | [0.89, 1.53] | 1.09 | [0.69, 1.70] | 1.25 | [0.96, 1.63] | | 1.25 | [0.95, 1.66] | 0.05 | [-0.06, 0.16] | 0.02 | [-0.08, 0.12] |
|  | 2010, δ_2_ | 0.75^*^ | [0.59, 0.95] | 0.84 | [0.55, 1.29] | 1.25^✝^ | [0.99, 1.58] | | 1.09 | [0.85, 1.41] | 0.05 | [-0.05, 0.15] | 0.00 | [-0.09, 0.09] |
|  | 2012, δ_3_ | 0.81^✝^ | [0.63, 1.03] | 0.76 | [0.49, 1.16] | 0.98 | [0.77, 1.25] | | 1.14 | [0.88, 1.47] | 0.03 | [-0.07, 0.13] | 0.00 | [-0.08, 0.09] |
|  | 2014, δ_3_ | 0.68^**^ | [0.54, 0.86] | 0.80 | [0.53, 1.18] | 0.96 | [0.76, 1.20] | | 0.98 | [0.77, 1.25] | 0.08 | [-0.02, 0.17] | 0.06 | [-0.03, 0.14] |
|  | 2016, δ_4_ | 0.70^**^ | [0.56, 0.88] | 0.74 | [0.50, 1.11] | 1.31^*^ | [1.04, 1.63] | | 1.55^***^ | [1.23, 1.96] | 0.29^***^ | [0.20, 0.38] | 0.19^***^ | [0.11, 0.27] |
| VP | L2 residual variance, var(*u*_0j_) | 0.09 |  | 0.00 |  | 0.07 |  | | 0.06 |  | 0.80 |  | 0.63 |  |

*Note*s. The coefficient estimates corresponding to the hypotheses are highlighted in yellow; “VP” = Variance parameters.

^***^*p* < .001, ^**^*p* < .01, ^*^*p* < .05, ^✝^*p* < .01

# Study 3: Preregistered Power Analysis

We conducted a power analysis using G*Power (Faul et al., 2007). The power analysis revealed that a sample size of *N* = 1,034 participants was needed to detect a significant small-sized effect of Black poverty on interracial anxiety (i.e., the *a* path) of *f* = .10 (⬄ η^2^_p_ ≈ .01 ⬄ *r* ≈ .10), while using a two-tailed test with α = .05, with a power of 1 – β = .8946. This meant that *N* = 1,034 participants were needed to detect three significant small-sized indirect effects (i.e., *a* × *b*) with an overall power of .8946^2^, that is, a power of .80.

# Study 3. Structural Equation Model

|  | $\text{anxiety}_{\text{ij}}\text{ = }\text{β}_{\text{0}}\text{ + }\text{β}_{\text{a}}\text{ × }\text{Black poverty}_{\text{ij}}\text{ + }{\text{ }\text{α}}_{\text{j}}\text{ }{\text{+ }\text{e}}_{\text{ij}}$ | (4a) |
| --- | --- | --- |
|  | $\text{threat}_{\text{ij}}\text{ = }\text{β}_{\text{0}}\text{ + }\text{β}_{\text{c'}}\text{ × }\text{Black poverty}_{\text{ij}}\text{ + }\text{β}_{\text{b}}\text{ × }\text{anxiety}_{\text{ij}}{\text{+ }\text{α}}_{\text{j}}\text{ }{\text{+ }\text{e}}_{\text{ij}}$ | (4b) |
|  | $\text{internal}_{\text{ij}}\text{ = }\text{β}_{\text{0}}\text{ + }\text{β}_{\text{d1'}}\text{ × }\text{Black poverty}_{\text{ij}}\text{ + }\text{β}_{\text{b}}\text{ × }\text{threat}_{\text{ij}}{\text{+ }\text{α}}_{\text{j}}\text{ }{\text{+ }\text{e}}_{\text{ij}}$ | (4c) |
|  | $\text{external}_{\text{ij}}\text{ = }\text{β}_{\text{0}}\text{ + }\text{β}_{\text{d2'}}\text{ × }\text{Black poverty}_{\text{ij}}\text{ + }\text{β}_{\text{b}}\text{ × }\text{threat}_{\text{ij}}{\text{+ }\text{α}}_{\text{j}}\text{ }{\text{+ }\text{e}}_{\text{ij}}$ | (4d) |

…*i* = 1, 2, …, *N* [participants], *j* = 1, 2, …, *K* [states], where *Black poverty*_j_ represents Black poverty (predictor), *anxiety*_ij_ represents interracial anxiety (mediator #1) , *threat*_ij_ represents identity threat (mediator #2), *internal*_ij_ represents internal attributions (outcome #1), *external*_ij_ represents external attributions (outcome #2), α_j_ represents the state fixed-effects, and *e*_ij_ represents the residuals.

**Figure S1**

*Study 3, H_4_ – Paths of Interest: Effects of the induction of Black poverty on internal attributions (upper part) and external attributions (lower part) of racial inequality, as mediated by interracial anxiety and identity threat while including our preregistered set of control variables: (gender, age, marital status, working status, education, log-transformed equivalized household income, and political orientation).*


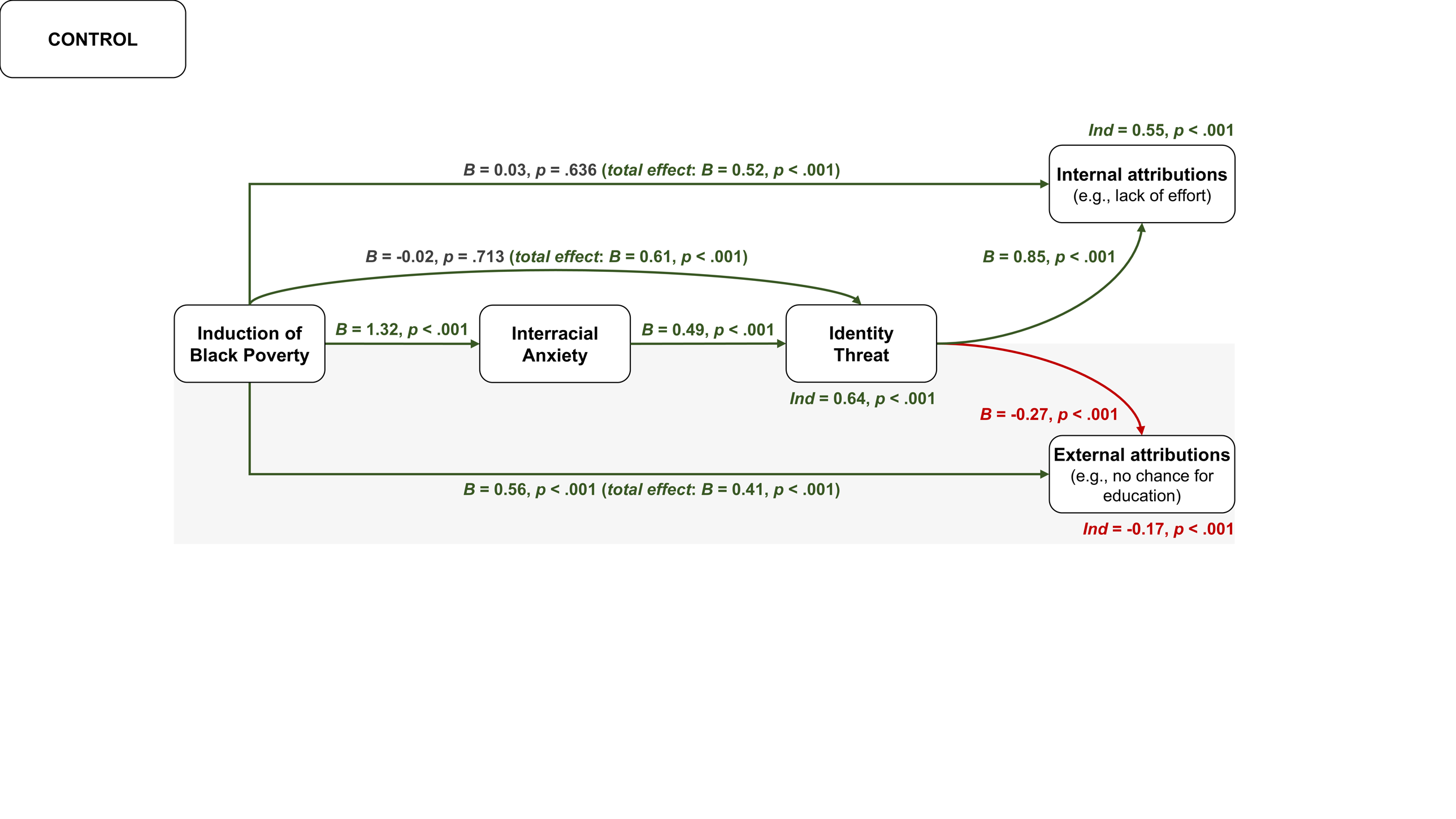


*Note.* Thick green lines represent positive effects; thick red lines represent negative effects; dashed grey lines represent null effects. Latent variables were used; however, for the sake of readability, we do not display the individual items or their loading factors. “*ind*” means indirect effect, namely, *B*_poverty→anxiety_ × *B*_anxiety→threat_ for the first one from the left, and *B*_poverty→anxiety_ × *B*_anxiety→threat_ × *B*_threat→attributions_ for the other two.

# Former Study 3.

The following experiment, designed to manipulate Black poverty, was included in the original manuscript. It was eventually discarded, as the Editor of the manuscript and one reviewer noted that it lacked proper random assignment and failed to clearly isolate Black poverty as the causal variable. This experiment was preregistered (https://osf.io/9mqh2).

**Method**

***Sample***

A preregistered power analysis revealed that 716 participants were needed to detect a mediation with small-sized paths of η^2^_p_s = .01, with *power* = .90 and α = .05.^[[1]](#footnote-1)^ We recruited a sample of 716 White Americans using CloudResearch’s MicroBatch tool, using two interlocked quotas to match U.S. demographics in terms of gender and education. After excluding five participants incorrectly identified as White by CloudResearch, our sample comprised 712 participants (**Table S6** presents the sample characteristics).

***Procedure and Variables***

All variables in the experiment used a response scale ranging from 1 = *Strongly disagree* to 7 = *Strongly agree* (**Table S7** presents the descriptive statistics). The procedure was divided into three steps.

**Step #1 Baseline Measures.**

***Attributions of Racial Inequality.*** We adapted Cozzarelli et al.’s (2001) Attributions for Poverty Scale, asking participants why “Black Americans have on average worse jobs, income, and housing than White Americans.” Three items measured *internal* attributions (i.e., “lack of effort,” “lack of thrift,” and “no attempt at self-improvement” by Black Americans), and three items measured *external* attributions (i.e., “denied an equal chance for education,” “not enough opportunities,” and “discrimination” faced by Black Americans).

***Interracial Anxiety.*** We adapted Stephan and Stephan’s (1985) measure of intergroup anxiety, asking participants to report the feelings they usually experience “when interacting with Black people,” specifically “tense,” “uneasy,” “bothered,” and “nervous.”

**Step #2 Induction of Black Poverty.** After completing the baseline measures, participants read a text instructing them to recall a place with high Black poverty. We chose this approach over manipulating bogus poverty rates to ensure a more valid induction, exposing participants to ecological cues indicative of Black poverty.

*In this task, we would like you to remember a place you have visited in the U.S. where many of the Black residents seemed to earn little income. This place could be a county, a city, a town or a neighborhood, and you may have stayed in this place for a couple of days, weeks, or even months. The important thing is that the Black residents of this place seemed to be poor. You may have realized this based on the way Black residents of this place looked, talked, or acted. Or maybe some Black residents approached you to ask for money. In any case, you could tell that many of the Black residents of this place were likely below the poverty line.*

Then, in two open-ended questions, participants were asked to describe the place in a few sentences (e.g., “Where was this place?”; “What did this place look like?”), and their thoughts and feelings while there (e.g., “What did you think of this place?”; “How did you feel there?).

**Step #3 Test Measures.** Following the induction, participants completed the same measures of attributions and interracial anxiety used in Step #1, except the items did not refer to *Black Americans*, but to *the Black residents of the place participants had visited*.

**Results**

***H_2a-b_. Black Poverty ^+^→ Internal/External Attributions of Racial Inequality***

As a first step, we tested Black poverty as a within-participant predictor of internal and external attribution score (in two separate models). Regression equations are presented at the end of the study, p. 6. **Table S8** presents the full results and **Figure S2** shows the focal effects.

Consistent with H_2a_, our analysis revealed that participants used more internal attributions to explain racial inequality among the residents of the place with high Black poverty than among Americans in general, *B* = 0.16 [0.09, 0.23], *p* < .001, η^2^_p_ = .03 (*c1* path). Regarding H_2b_, our analysis revealed that the negative effect of Black poverty on external attributions of racial inequality was only “marginal,” *B* = -0.06 [0.13, 0.01], *p* = .072, η^2^_p_ = .005 (*c2* path).

***H_4_. Black Poverty ^+^→ Interracial Anxiety ^+^→ Internal/External Attributions.***

As a second step, we tested whether the effects of Black poverty on attributions were mediated by an increase in interracial anxiety. We first tested Black poverty as a within-participant predictor of interracial anxiety (*a* path), and then repeated the analysis testing the effects of Black poverty on attribution while including interracial anxiety as a covariate (*b*/*c′* paths). As preregistered, we calculated the indirect effects using the percentile bootstrap method with 10^4^ resamples (Yzerbyt et al., 2018). Regression equations are presented at the end of the study. **Table S8** presents the full results and **Figure S3** shows the path of interest.

Our analysis revealed that participants experienced more interracial anxiety in the place with high Black poverty than with Black Americans in general, *B* = 1.18 [1.06, 1.30], *p* < .001, η^2^_p_ = .34 (*a* path). Consistent with H_4_, interracial anxiety positively predicted internal attributions, *B* = 0.12 [0.07, 0.17], *p* < .001, η^2^_p_ = .03 (*b1* path), explaining 88.2% of the effect of Black poverty on internal attributions, *ind* = 0.14 [0.07, 0.21], *p* < .001 (*a* × *b1* path). Inconsistent with H_4_, interracial anxiety neither predicted external attributions, *B* = 0.02 [-0.02, 0.07], *p* = .347 (*b2* path), nor mediated the effect of Black poverty on external attribution, *ind* = 0.03 [-0.06, 0.12], *p* = .312 (*a* × *b2* path).

***Supplementary Analysis***: ***Control Variables.***

We repeated the main analysis while controlling for the same individual variables used in Studies 1-2, excluding marital status (erroneously omitted from the survey). The links observed in the main analysis remained the same when controlling for our set of control variables: (i) Black poverty positively predicted internal attributions, *B* = 1.13 [0.09, 0.18], *p* < .001, η^2^_p_ = .03, but only negatively predicted external attributions at the α = .10 level, *B* = -0.06 [-0.13, 0.01], *p* = .077, η^2^_p_ = .004; (ii) Black poverty positively predicted interracial anxiety, *B* = 1.19 [1.07, 1.31], *p* < .001, η^2^_p_ = .35, which mediated the effect of Black poverty on internal attributions, *ind* = 0.14 [0.07, 0.20], *p* < .001, but not on external attribution, *ind* = 0.03 [0.02, 0.08], *p* < .001, *p* = .236 (**Table S9** presents the full results).

______________________

**Regression Equations for the Models Testing H_2a-b_**

|  | $\text{∆}_{\text{iatt}_{\text{i}}}\text{ = }\text{c1}\text{ }{\text{+}\text{ e}}_{\text{i}}$ | $\text{∆}_{\text{eatt}_{\text{i}}}\text{ = }\text{c2}\text{ }\text{+}\text{ e}_{\text{i}}$ | (4) |
| --- | --- | --- | --- |

…*i* = 1, 2, …, *N* [participants], where *iatt*_i_ represents the internal attribution score, and *eatt*_i_ represents the external attribution score, and where “∆” designates the difference between the pre- and post-induction measures, and *e*_i_ is the error term.

**Regression Equations for the Models Testing *H_3_***

|  | $\text{∆}_{\text{anx}_{\text{i}}}\text{ = }\text{a}\text{ }{\text{+}\text{ e}}_{\text{i}}$ | | (5) |
| --- | --- | --- | --- |
|  | $\text{∆}_{\text{iatt}_{\text{i}}}\text{ =}\text{ }\text{c1'}\text{ + }\text{b1}\text{ × }\text{∆}_{\text{anx}_{\text{i}}}+\text{d1}\text{ × }\frac{\bar{\text{Σ}_{\text{anx}_{\text{i}}}}}{\text{2}}{\text{ + }\text{e}}_{\text{i}}$ | $\text{∆}_{\text{eatt}_{\text{i}}}\text{ =}\text{ }\text{c2'}\text{ }\text{+ }\text{b2}\text{ ×}\text{ }\text{∆}_{\text{anx}_{\text{i}}}+\text{d2}\text{ × }\frac{\bar{\text{Σ}_{\text{anx}_{\text{i}}}}}{\text{2}}{\text{ }\text{+ }\text{e}}_{\text{i}}$ | (6) |

…*i* = 1, 2, …, *N* [participants], where *anx*_i_ represents interracial anxiety, *iatt*_i_ represents the internal attribution score, and *eatt*_i_ represents the external attribution score, and where “∆” designates the difference between the pre- and post-induction measures, and $\bar{\Sigma}$ designate the mean-centered sum of these measures.

# Table S6. Former Study 3: Sample Characteristics

| Proportion of men | .49 [.46, .53] |
| --- | --- |
| Mean age | 42.2 [41.2, 43.2] |
| Proportion of married participants | n/i |
| Proportion in the workforce | .76 [.73, .79] |
| Education (Proportion of college-graduated participants) | .36 [.33, .40] |
| Mean annual equalized income (in US$ 10^4^) | 5.41 [5.13, 5.70] |
| Mean political orientation (1 = *Very liberal* to 5 = *Very conservative* | 3.70 [3.57, 3.84] |

*Note*. To determine income from the closed-ended household income items, we took the midpoint of the income band (for all but the last categories), or derived the value from Hout’s (2004) Pareto curve-based formula (for the last category); to adjust for household size, we used OECD’s (2009) equivalization formula (i.e., $\text{income}_{\text{adj}}\text{= }\text{income}\text{ ÷ }\sqrt{\text{household size}}$); “n/i” means “not included” (the variable was erroneously omitted from the survey).

# Table S7. Former Study 3 – Descriptive Statistics

*Reliability coefficients, means/standard deviations, and correlation matrix for the focal variables*

|  | | *Descriptive statistics* | | | Zero-order *correlation matrix.* | | | | | |
| --- | --- | --- | --- | --- | --- | --- | --- | --- | --- | --- |
|  |  | α | *M* | *SD* | 1 | 2 | 3 | 4 | 5 | 6 |
| Baseline measures | Internal attributions of racial inequality | .92 | 3.41 | 1.70 | 1.00 |  |  |  |  |  |
|  | External attributions of racial inequality | .92 | 4.50 | 1.78 | -0.64^***^ | 1.00 |  |  |  |  |
|  | Interracial anxiety | .97 | 2.11 | 1.29 | 0.31^***^ | -0.08^*^ | 1.00 |  |  |  |
| Post-induction measures | Internal attributions of racial inequality | .94 | 3.57 | 1.79 | 0.84^***^ | -0.61^***^ | 0.30^***^ | 1.00 |  |  |
|  | External attributions of racial inequality | .92 | 4.44 | 1.84 | -0.60^***^ | 0.88^***^ | -0.08^*^ | -0.58^***^ | 1.00 |  |
|  | Interracial anxiety | .96 | 3.29 | 1.87 | 0.31^***^ | -0.14^***^ | 0.51^***^ | 0.40^***^ | -0.12^**^ | 1.00 |

^***^*p* < .001, ^**^*p* < .01, ^*^*p* < .05

# Table S8. Former Study 3 – Full Results

*Bs and 95% CIs* *from the models testing the effects of Black poverty on attributions (H_2a_ and H_2b_), as mediated by interracial anxiety (H_4_)*

|  | *a* path | | *c1* path | | *b1* & *c1*′ path | | *c2* path | | *b2* & *c2*′ path | |
| --- | --- | --- | --- | --- | --- | --- | --- | --- | --- | --- |
|  | Interracial anxiety ($\text{∆}_{\text{anx}_{\text{i}}}$) | | Internal attributions  ($\text{∆}_{\text{iatt}_{\text{i}}}$) | | | | External attributions ($\text{∆}_{\text{e}\text{att}_{\text{i}}}$) | | | |
|  | *B* | 95% | *B* | 95% | *B* | 95% | *B* | 95% | *B* | 95% |
| Black Poverty (*a*, *c*, and *c’* paths) | 1.18^***^ | [1.06, 1.30] | 0.16^***^ | [0.09, 0.23] | 0.02 | [-0.07,0.11] | -0.06^✝^ | [-0.13, 0.01] | -0.09^*^ | [-0.17,-0.00] |
| Interracial anxiety (*b* paths) |  |  |  |  | 0.12^***^ | [0.07, 0.17] |  |  | 0.02 | [-0.02, 0.07] |
| Average interracial anxiety (*d paths*) |  |  |  |  | 0.04 | [-0.01, 0.10] |  |  | 0.01 | [-0.04, 0.06] |

*Note*: The coefficient estimates corresponding to the hypotheses are highlighted in yellow. We used Montoya and Hayes’s (2017) centering method, adding the mean-centered average of the mediator variables to the regression equation (i.e., *d*, which is not be interpreted).

^***^*p* < .001, ^**^*p* < .01, ^*^*p* < .05

**Figure S2. Former Study 3 – Total Effects**

*Internal attribution score (left panel; H_2a_) and external attribution score (right panel; H_2b_) among White participants, as a function of the induction of Black poverty.*


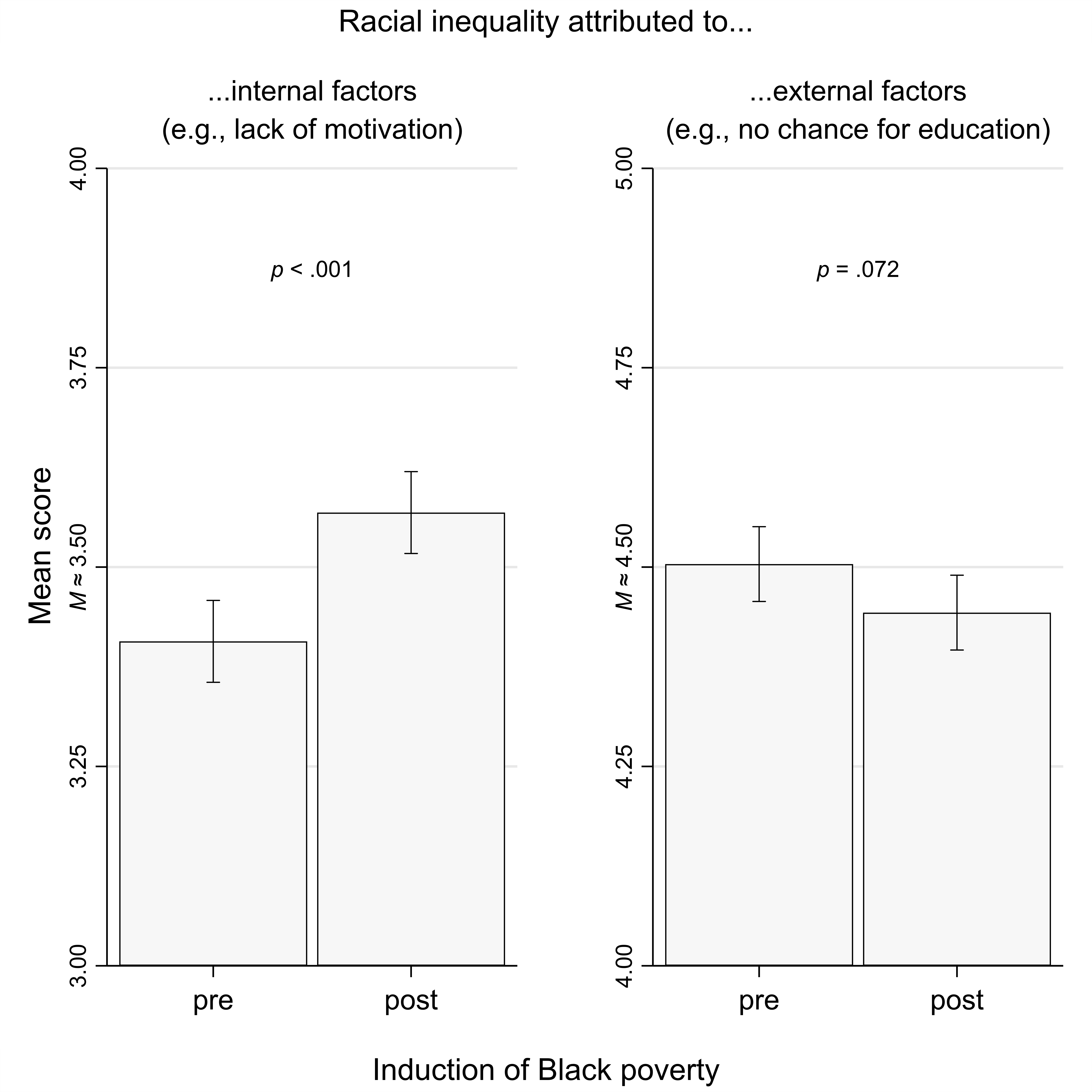


*Notes*. The response scale could range from 1 = *Strongly disagree* to 7 = *Strongly agree*; error bars represent 95% CIs.

**Figure S3. Former Study 3 – Path of Interest for the Mediation Model**

*Effects of the induction Black poverty (within participant) on internal attributions (upper part) and external attributions (lower part) of racial inequality, as mediated by interracial anxiety among White participants.*

*
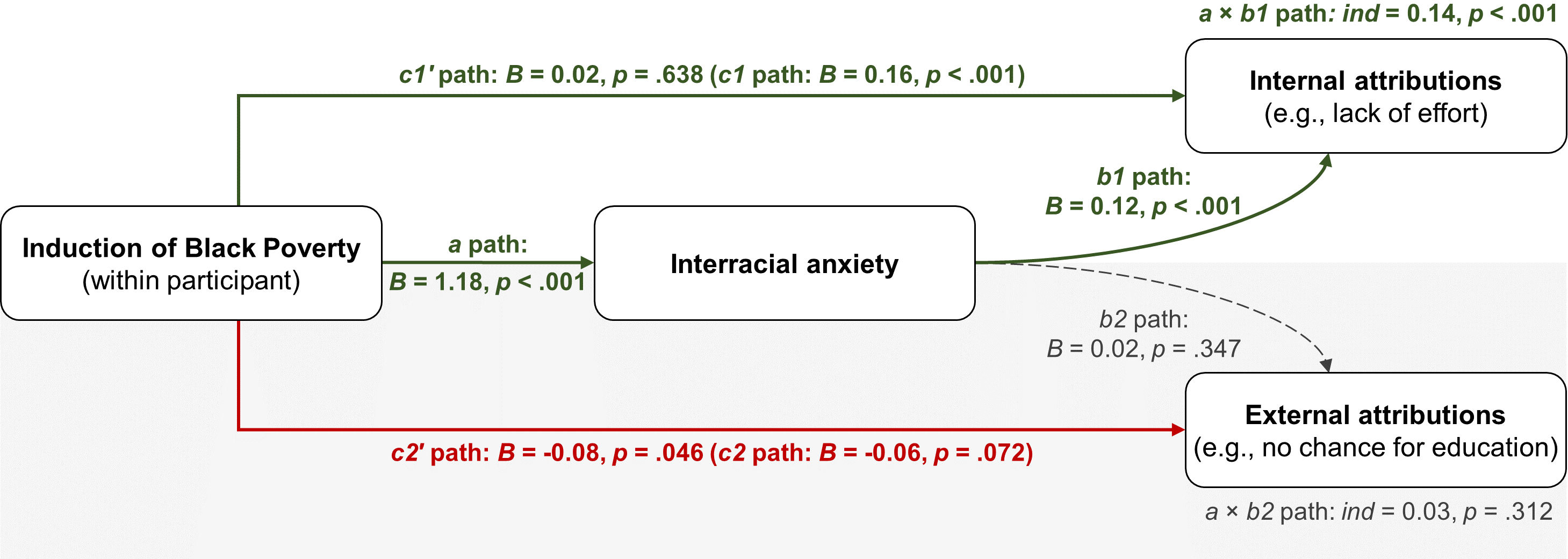
Notes*. Thick green lines represent positive effects; thick red lines represent negative effects; dashed grey lines represent null effects; total effects are given in parentheses.

# Table S9. Former Study 3 – Full Results With Control Variable

*Bs/95% CIs from the models testing the effects of Black poverty on attributions, as mediated by interracial anxiety*

|  | *a* path | | *c1* path | | *b1* & *c1*′ path | | *c2* path | | *b2* & *c2*′ path | |
| --- | --- | --- | --- | --- | --- | --- | --- | --- | --- | --- |
|  | Interracial anxiety ($\text{∆}_{\text{anx}_{\text{i}}}$) | | Internal attributions  ($\text{∆}_{\text{iatt}_{\text{i}}}$) | | | | External attributions  ($\text{∆}_{e\text{att}_{\text{i}}}$) | | | |
|  | *B* | 95% | *B* | 95% | *B* | 95% | *B* | 95% | *B* | 95% |
| Black Poverty (*a*, *c*, and *c’* paths) | 1.19^***^ | [1.07,1.31] | 0.16^***^ | [0.09,0.24] | 0.03 | [-0.06,0.12] | -0.06 | [-0.13,0.01] | -0.09^*^ | [-0.17,-0.00] |
| Interracial anxiety (*b* paths) |  |  |  |  | 0.11^***^ | [0.07,0.16] |  |  | 0.02 | [-0.02,0.07] |
| Average interracial anxiety (*d paths*) |  |  |  |  | 0.05 | [-0.00,0.11] |  |  | -0.01 | [-0.06,0.05] |
| Gender, *B*_1_ | -0.17^**^ | [-0.29,-0.05] | -0.05 | [-0.12,0.03] | -0.02 | [-0.10,0.05] | 0.03 | [-0.03,0.10] | 0.04 | [-0.03,0.10] |
| Age, *B*_2_ | 0.1 | [-0.03,0.23] | 0 | [-0.07,0.08] | -0.01 | [-0.08,0.07] | 0 | [-0.07,0.07] | 0 | [-0.07,0.07] |
| Employment status, *B*_3_ | -0.02 | [-0.15,0.11] | -0.01 | [-0.09,0.07] | -0.01 | [-0.08,0.07] | 0 | [-0.07,0.07] | 0 | [-0.07,0.07] |
| Education, *B*_4_ | -0.05 | [-0.19,0.09] | -0.02 | [-0.10,0.07] | -0.02 | [-0.11,0.06] | 0.03 | [-0.05,0.11] | 0.03 | [-0.05,0.11] |
| Log equalized income, *B*_5_ | 0.07 | [-0.07,0.21] | 0 | [-0.09,0.09] | -0.01 | [-0.09,0.08] | 0.05 | [-0.03,0.13] | 0.05 | [-0.03,0.13] |
| Political orientation, *B*_6_ | 0.09 | [-0.04,0.21] | -0.03 | [-0.10,0.05] | -0.05 | [-0.13,0.02] | 0.08^*^ | [0.01,0.15] | 0.08^*^ | [0.01,0.15] |

*Note*: The coefficient estimates corresponding to the hypotheses are highlighted in yellow; all control variables were standardized.

^***^*p* < .001, ^**^*p* < .01, ^*^*p* < .05

# Supplementary References

Blake, K. R., & Gangestad, S. (2020). On attenuated interactions, measurement error, and statistical power: guidelines for social and personality psychologists. *Personality and Social Psychology Bulletin, 46*(12), 1702-1711.

Brysbaert, M. (2019). How many participants do we have to include in properly powered experiments? A tutorial of power analysis with reference tables. *Journal of Cognition, 2*(1), 16.

Cozzarelli, C., Wilkinson, A. V., & Tagler, M. J. (2001). Attitudes toward the poor and attributions for poverty. *Journal of Social Issues, 57*(2), 207-227.

Faul, F., Erdfelder, E., Lang, A.-G., & Buchner, A. (2007). G* Power 3: A flexible statistical power analysis program for the social, behavioral, and biomedical sciences. *Behavior Research Methods, 39*(2), 175-191.

Gignac, G. E., & Szodorai, E. T. (2016). Effect size guidelines for individual differences researchers. *Personality and Individual Differences, 102*, 74-78.

Hout, M. (2004). *Getting the most out of the GSS income measures*. National Opinion Research Center Chicago

LeBeau, B. (2019). *Simglm: Simulate models based on the generalized linear model (R package version 0.8.0)*. CRAN

Midi, H., Sarkar, S. K., & Rana, S. (2010). Collinearity diagnostics of binary logistic regression model. *Journal of Interdisciplinary Mathematics, 13*(3), 253-267.

Montoya, A. K., & Hayes, A. F. (2017). Two-condition within-participant statistical mediation analysis: A path-analytic framework. *Psychological Methods, 22*(1), 6-27.

OECD. (2009). What are equivalence scales? In *OECD project on income distribution and poverty*. OECD Publishing

Sommet, N., Weissman, D., Cheutin, N., & Elliot, A. J. (2023). How many participants do i need to test an interaction? Conducting an appropriate power analysis and achieving sufficient power to detect an interaction. *Advances in Methods and Practices in Psychological Science, 6*.

Stephan, W. G., & Stephan, C. W. (1985). Intergroup anxiety. *Journal of Social Issues, 41*(3), 157-175.

U.S. Census Bureau. (2022). *Explore Census Data*. <https://data.census.gov/cedsci>.

Yzerbyt, V., Muller, D., Batailler, C., & Judd, C. M. (2018). New recommendations for testing indirect effects in mediational models: The need to report and test component paths. *Journal of Personality and Social Psychology, 115*(6), 929-943.

1. We conducted a power analysis using G*Power (Faul et al., 2007). The power analysis revealed that a sample size of *N* = 716 participants were needed to detect a significant small-sized effect of Black poverty on interracial anxiety (i.e., the *a* path) of *f* = .10 (⬄ η^2^_p_ ≈ .01 ⬄ *r* ≈ .10), while assuming a between-measurement correlation of *ρ* = .25 (a very conservative estimate; Brysbaert, 2019), and using a two-tailed test with α = .05, with a power of 1 – β = .90^1/3^. This meant that *N* = 716 participants were needed to detect three significant small-sized *a*, *b1*, and *b2* paths (the three critical paths) with an overall power of .90^1/3^ to the 3^rd^ power, that is, a power of .90. [↑](#footnote-ref-1)
